# mTORC1 Maintains the Tumorigenicity of SSEA-4^+^ High-Grade Osteosarcoma

**DOI:** 10.1038/srep09604

**Published:** 2015-04-08

**Authors:** Wu Zhang, Meng-Lei Ding, Jia-Nian Zhang, Jian-Ru Qiu, Yu-Hui Shen, Xiao-Yi Ding, Lian-Fu Deng, Wei-Bin Zhang, Jiang Zhu

**Affiliations:** 1 State Key Laboratory for Medical Genomics, Shanghai Institute of Hematology and Collaborative Innovation Center of Hematology, Rui-Jin Hospital affiliated to Shanghai Jiao-Tong University School of Medicine, Shanghai 200025, People's Republic of China; 2 Division of Orthopedics and Shanghai Institute of Traumatology and Orthopaedics, Shanghai 200025, People's Republic of China; 3 Shanghai Institute of Digestive Surgery, Shanghai 200025, People's Republic of China; 4 Department of Radiology, Rui-Jin Hospital, Shanghai 200025, People's Republic of China; 5 Collaborative Innovation Center of Systems Biomedicine, Shanghai 200025, People's Republic of China

## Abstract

Inactivation of p53 and/or Rb pathways restrains osteoblasts from cell-cycle exit and terminal differentiation, which underpins osteosarcoma formation coupled with dedifferentiation. Recently, the level of p-S6K was shown to independently predict the prognosis for osteosarcomas, while the reason behind this is not understood. Here we show that in certain high-grade osteosarcomas, immature SSEA-4^+^ tumor cells represent a subset of tumor-initiating cells (TICs) whose pool size is maintained by mTORC1 activity. mTORC1 supports not only SSEA-4^+^ cell self-renewal through S6K but also the regeneration of SSEA-4^+^ TICs by SSEA-4^−^ osteosarcoma cell dedifferentiation. Mechanistically, active mTORC1 is required to prevent a likely upregulation of the cell-cycle inhibitor p27 independently of p53 or Rb activation, which otherwise effectively drives the terminal differentiation of SSEA-4^−^ osteosarcoma cells at the expense of dedifferentiation. Thus, mTORC1 is shown to critically regulate the retention of tumorigenicity versus differentiation in discrete differentiation phases in SSEA-4^+^ TICs and their progeny.

Osteosarcoma represents a type of highly aggressive bone tumor prevalent in adolescents and is characterized by composite genetic defects. Early observations pointed to genetic defects in the *Rb* or *p53* pathway as driving events behind tumorigenesis^1^,^2^. Accordingly, recent studies have indicated that in all osteosarcoma cases, the p53 pathway is functionally defective^3^. More than 70% of sporadic osteosarcoma cases harbor genetic abnormalities involving the Rb pathway^4^. Although the targeted deletion of *p53* but not *Rb* alone caused murine osteosarcomas to develop, the simultaneous deletion of *p53* and *Rb* significantly accelerated tumorigenesis^4^,^5^,^6^, indicating that inactivation of these two pathways cooperates to drive malignant transformation^7^,^8^,^9^,^10^.

The dedifferentiation process essentially underlies the genesis of osteosarcoma that is marked with mesenchymal immaturity, as the targeted-deletion of *p53* or both *p53* and *Rb* in committed or mature osteoblasts rather than in mesenchymal stem cells (MSCs) results in osteosarcoma in mouse models^4^,^11^,^12^,^13^. In agreement with this, recent studies demonstrate that *p53* or *Rb* deficiency activates the dedifferentiation potential of many types of somatic cells^10^,^14^. Since a prior cell-cycle exit is required for immature osteosarcoma cells to undergo the terminal maturation that likely eliminates their tumorigenicity^15^,^16^, and p53 and Rb pathways share the critical activity of restraining cell-cycle progression, defects in the p53 or/and Rb pathway(s) may confer dedifferentiation potential to osteosarcoma cells largely by reinstituting their entry into the cell cycle from the post-mitotic state^17^.

On the other hand, cases of osteosarcoma can be highly heterogeneous in terms of their clinical prognosis. With the introduction of combined systemic chemotherapy plus surgery, approximately 60–70% of newly-diagnosed osteosarcoma patients actually achieve long-term survival^18^, whereas the remaining cases are chemo-resistant and prone to metastasize, thus constituting a high-grade subgroup^19^,^20^. Few studies have addressed this clinical heterogeneity^21^. A recent study did report that the level of p-S6, an indictor of mTOR activity, positively correlated with poor prognosis in osteosarcoma^22^. In parallel, a phase I–II clinical trial of mTOR inhibitors on a panel of pediatric tumors revealed a plausible therapeutic benefit in a few advanced osteosarcoma cases^23^,^24^,^25^. More recently, use of a combination of multi-kinase inhibitors restrained the growth of osteosarcoma cell lines *in vitro* and *in vivo*, which was accompanied by a reduction in the activities of mTORC1 and mTORC2^26^. Nevertheless, the precise role mTOR plays in the regulation of the malignant stemness of osteosarcoma cells remains unclear.

Interestingly, all osteosarcoma cases, including high-grade ones, are pathologically characterized by the spontaneous formation of osteoid, an unmineralized pre-bone structure produced largely by mature osteocytes. This seems to indicate that putative osteosarcoma tumor-initiating cells (TICs) retain the potential to undergo terminal osteogenic maturation *in vivo*. In this study, we explore which cellular mechanisms control terminal differentiation versus the maintenance of malignant immaturity associated with high-grade osteosarcoma, which may shed potential light on the design of new therapeutic regimens for this currently-incurable malignancy.

## Results

### SSEA-4^+^ osteosarcoma cells represent a subset of xenografting-TICs

**Figure 1:**
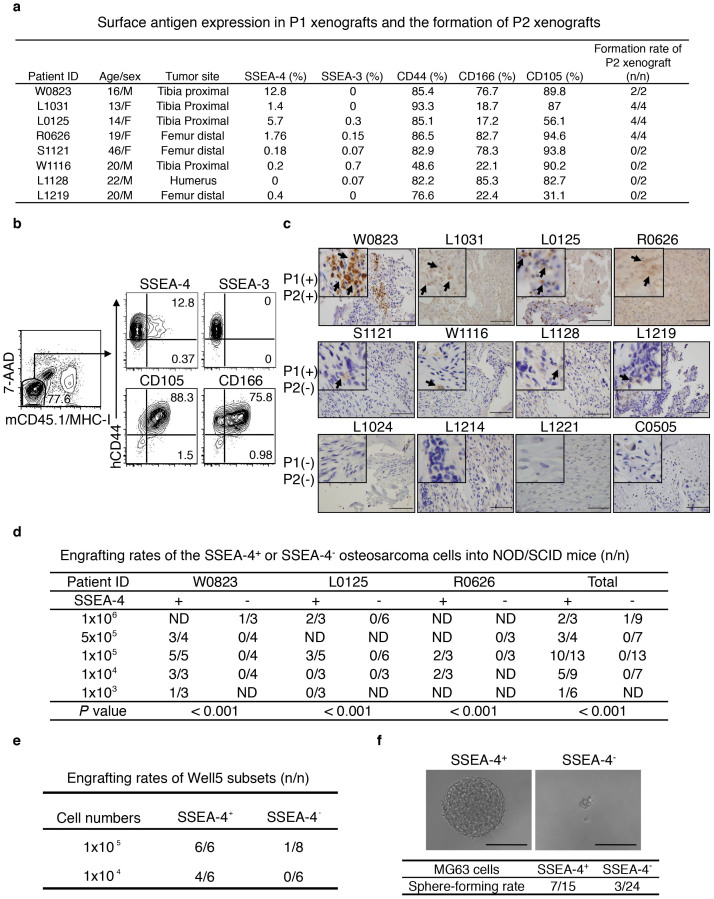
SSEA-4 Labels Xenografting-TICs Present in a Minority of Human Osteosarcoma Cases. (a) The frequency of SSEA-4^+^ cells in P1 xenografts correlates with xenograft-formation potential in secondary recipients. (b) Live 7-AAD^−^ and murine CD45^+^/MHC-I^+^-excluded osteosarcoma xenograft cells were analyzed for expression of the indicated antigens by flow cytometry. (c) Primary osteosarcoma samples that generate both P1 and P2 xenografts show higher SSEA-4-staining intensity than those that produced only P1 xenografts. Primary specimens that produced no P1 xenografts stained negatively for SSEA-4. Immunohistochemical staining signals for SSEA-4 are indicated by arrows. Scale bar represents 100 μm. (d) Engrafting efficiencies of SSEA-4^+^ or SSEA-4^−^ cells freshly isolated from three individual tumorigenic xenografts (P2 to P5). (e) Xenografting efficiency of SSEA-4^+^ or SSEA-4^−^ osteosarcoma cells isolated from *in vitro*-cultivated Well5 cells (*P* < 0.05). (f) Sphere-forming rate of SSEA-4^+^ or SSEA-4^−^ cells isolated from *in vitro* cultivated osteosarcoma MG63 cells (*P* < 0.05). Microscopic inspection of representative tumor-sphere or cellular debris is shown in the upper panel.

To identify putative TICs for osteosarcoma, we subcutaneously inoculated minced human osteosarcoma tissue (typically ≤ 3 × 3 × 1.5 mm^3^) into NOD/SCID mice. Eight of 21 primary specimens generated passage 1 (P1) xenografts wherein the retention of osteosarcoma identity was evidenced by the presence of osteoid formations (Supplementary Fig. 1a). Interestingly, upon the secondary inoculation of 1–10 × 10^6^ huCD44-labeled P1 cells per recipient, only the four P1 xenografts containing SSEA-4^+^ cells (>1%) but not others generated P2 xenografts (Fig. 1a, b). Likewise, as assayed by immunohistochemical staining, only SSEA-4^+^ cells-containing primary samples generated P1 xenografts, with SSEA-4^+^ cells being more frequently detected in those also producing P2 xenografts (Fig. 1c and data not shown). Immunofluorescent co-staining of tissue sections and FISH assays on sorted SSEA-4^+^ cells showed that the expression of p53 or/and Rb was absent in most SSEA-4^+^ cells, thus discriminating them from nonmalignant stromal cells (Supplementary Fig. 1b). As this secondary xenografting rate was likely determined by the frequency of TIC within the P1 xenografts, we assumed that SSEA-4 could be used as a xenografting-TIC-enriching biomarker.

To verify the co-purification of SSEA-4^+^ cells with tumorigenicity, we then prospectively sorted huCD44-labeled SSEA-4^+^ or SSEA-4^−^ cells from the P2 to P5 xenografts of 3 clinical specimens (namely W0823, L1031 and R0626) using fluorescence-activated cell sorting (FACS) and inoculated the serially-diluted tumor cells into NOD/SCID mice. As expected, tumorigenicity was significantly enriched in SSEA-4^+^ cells compared to SSEA-4^−^ cells (Fig. 1d). As expected, orthotopic injection of 1 × 10^4^ SSEA-4^+^ but not SSEA-4^−^ W0823-derived osteosarcoma cells into mouse femurs or tibias produced the characteristic osteosarcoma (Supplementary Fig. 1c). Nevertheless, these SSEA-4^+^ cell-containing xenografts hardly expressed any Stro-1, CD117, ABCG2 or CD133 that were shown to label TICs evident in several osteosarcoma cell lines (Supplementary Fig. 1d)^27^,^28^, nor did these xenografts contain any Hoechest-expelling side population (Supplementary Fig. 1e).

Actually, SSEA-4^+^ cells were also present within W0823-derived Well5 cells passaged *in vitro* and in osteosarcoma cell lines MG63 and U2OS cells, but not Saos-2 cells (Supplementary Fig. 1f, g and data not shown)^29^. Both Well5 and MG63 cells possessed mesenchymal multipotency showing bi-differentiation potential towards osteogenic and adipocytic lineages (Supplementary Fig. 1h), indicating that SSEA-4^+^ TICs remain at an immature stage before osteoblastic commitment. Tumorigenic xenograft-forming or tumorsphere-forming assays of Well5 or MG63 cells confirmed that tumorigenicity was much more enriched in the SSEA-4^+^ cell fraction than in SSEA-4^−^ cells (Fig. 1e, f). Nevertheless, ISP-1, an inhibitor of SSEA-4 synthesis, did not reduce the tumorsphere-forming potential of MG63 cells (Supplementary Fig. 1i), indicating that SSEA-4 itself represents a biomarker rather than a functional regulator of malignant stemness.

### Frequency of SSEA-4^+^ TICs predicts prognosis

**Figure 2:**
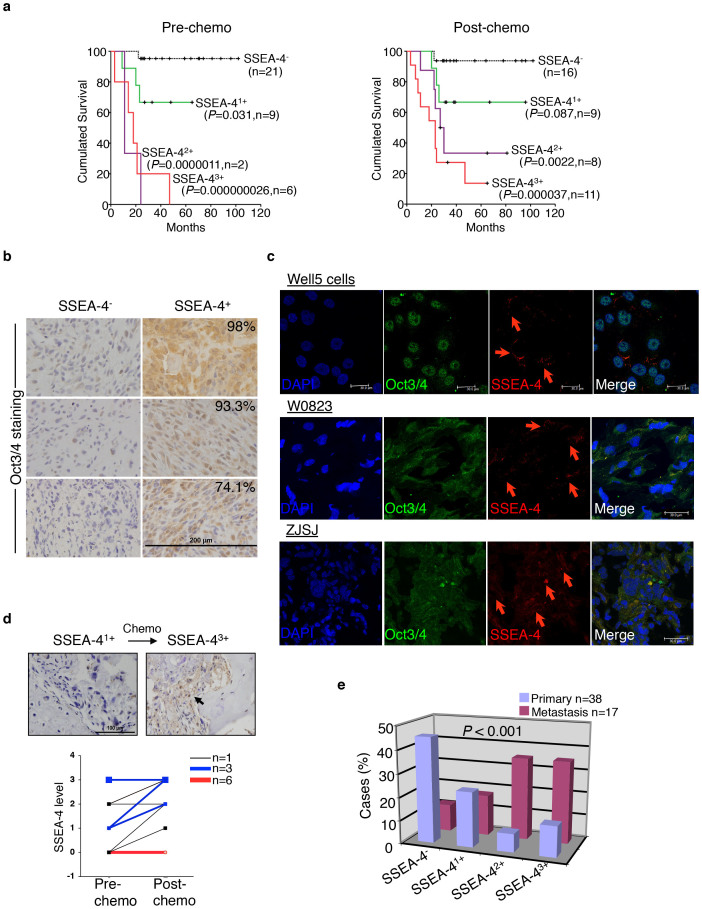
SSEA-4^+^ TICs are Responsible for the Clinical Progression of a Distinct Subtype of High-grade Osteosarcomas. (a) Overall survival of patients with discrete SSEA-4 staining intensities in osteosarcoma cases, as measured before (left panel) or after (right panel) the first round of chemotherapy. *P* values: compared with the SSEA-4^−^ subgroup; n: patient number. (b) Oct3/4 expression assayed on primary specimens of osteosarcoma that contained or did not contain SSEA-4^+^ TICs. Percentages of Oct3/4^+^ cells are shown in the upper-right corner. Six representative samples are shown. (c) Cryopreserved sections of SSEA-4^+^ xenografts were co-stained with fluorescent antibodies against Oct3/4 or SSEA-4. Three representative samples are shown. Red arrows indicate SSEA-4^+^ cells. (d) Upper panel, SSEA-4 staining before and after the first round of chemotherapy in one representative case. Bottom panel, SSEA-4-staining intensities of osteosarcoma samples from 19 patients measured before and after chemotherapy. Samples from the same patient are paired with lines. (e) Relative distribution rates of SSEA-4^−^, SSEA-4^1+^, SSEA-4^2+^, and SSEA-4^3+^ osteosarcoma samples among those obtained from primary or metastatic sites.

SSEA-4^+^ osteosarcoma cells were readily detectable only in a small fraction (8/21) of primary osteosarcoma specimens (Fig. 1c, d), which prompted us to test whether the osteosarcoma cases containing SSEA-4^+^ TICs represent a subtype of osteosarcoma distinct from the majority of SSEA-4^neg^ cases. To address this, we performed a retrospective analysis of a cohort of osteosarcoma cases collected over >10 years. Remarkably, the frequency of SSEA-4^+^ TICs alone, as indicated by immunohistochemical staining (arbitrarily determined as -, 1+, 2+, or 3+; see Supplementary Fig. 2a) before or after the first round of chemotherapy, correlated inversely with the overall-survival probabilities of patients (Fig. 2a). Strikingly, >90% of SSEA-4^neg^ cases but no SSEA-4^2+–3+^ cases survived more than 10 years after diagnosis. Notably, transcription factor Oct3/4, the expression of which marks MSCs and was reported in certain cultivated osteosarcoma cells to associate with a gain of tumorigenicity and probably reprogramming events^28^,^30^,^31^, was consistently and abundantly expressed in bulk tissue cells of SSEA-4^+2–3^ primary specimens (n = 26) wherein SSEA-4^+^ TICs constituted a small subfraction of Oct3/4^+^ osteosarcoma cells (Fig. 2b, c). In striking contrast, no Oct3/4 expression was detected in eight SSEA-4^neg^ cases examined (Fig. 2b, Supplementary Fig. 2b, c), thus indicating that SSEA-4^+^ TICs but not TICs from other subtypes of osteosarcoma have been reprogrammed to a highly immature status comparable to MSCs or even ES cells in certain molecular programs^32^.

In agreement with the assumption that SSEA-4^+^ TICs survived systemic chemotherapy, Well5 cells were much more resistant to multiple chemotherapeutic agents than SSEA-4^−^ Saos-2 osteosarcoma cells that were largely osteogenic lineage-committed (Supplementary Fig. 2d–f). Consistent with this, in all 11 samples initially diagnosed as SSEA-4^1+–3+^ osteosarcoma, chemotherapy enriched or maintained the frequency of SSEA-4^+^ cells (Fig. 2d); this was also confirmed by flow cytometric assay (Supplementary Fig. 2g). Actually, in 2 of the 8 cases initially diagnosed as SSEA-4^neg^, SSEA-4^+^ cells were detectable after chemotherapy (Fig. 2d), indicating either that a few SSEA-4^neg^ cases probably contained rare SSEA-4^+^ TICs or that SSEA-4^+^ TICs might arise in SSEA-4^neg^ cases through malignant evolution. Moreover, as predicted by the observation that a more grave prognosis was associated with SSEA-4^2+–3+^ cases and the notion that TICs seed metastasis, SSEA-4^2+–3+^ samples were more highly distributed within the metastatic lesions than within the primary sites (Fig. 2e).

### Mesenchymal differentiation of SSEA-4^+^ TICs parallels a gradual loss of tumorigenicity

**Figure 3:**
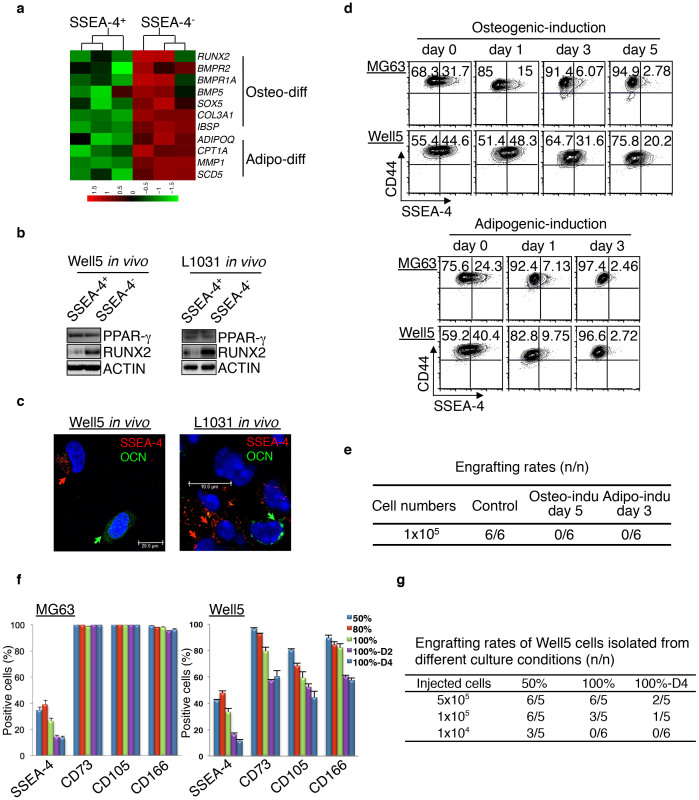
SSEA-4^+^ TICs Undergo Mesenchymal Differentiation to Generate SSEA-4^−^ Cells. (a) Heatmap showing expression levels of the signature gene sets that indicate the mesenchymal differentiation status of W0823-derived SSEA-4^+^ TICs or SSEA-4^−^ osteosarcoma cells. (b) Western blotting assays for the levels of PPAR-γ and RUNX2 in sorted SSEA-4^+^ or SSEA-4^−^ osteosarcoma cells growing *in vivo*. The cropped blots were run under the same experimental conditions. The full-length blots are included in Supplementary Figure 8. (c) Co-immunofluorescent staining and inspection of cytospun Well5-xenograft cells (left panel) or frozen sectioned (right panel) L1031-derived xenografts. The antibody against SSEA-4 was labeled with Alexa555 (Red) and the antibody against OCN was FITC-labeled (green). (d) SSEA-4^+^ cell frequency decreased in Well5 or MG63 cells undergoing osteogenic or adipogenic differentiation, as measured by flow cytometry. (e) Tumorigenic xenograft-forming rates of Well5 cells after prior treatment with DMSO or differentiation inducers for 3–5 days as in (d) (*P* = 0.002 for each induction versus control). (f) SSEA-4^+^ cell frequency measured for Well5 or MG63 cells under different culture conditions. The percentages indicate cell densities. One hundred %-D2 or 100%-D4 indicates an additional culture for 2 days or 4 days post-confluence. Results are shown as mean ± SD, n = 3. (g) TIC frequency measurement of Well5 cells obtained from different culture conditions as in (f). One hundred %-D4 versus 50%: *P* < 0.001. 100% versus 50%: *P* = 0.034.

The formation of osteoids observed in cases of high-grade osteosarcoma indicates that even highly dedifferentiated immature SSEA-4^+^ TICs might undergo spontaneous osteogenic maturation, although at a significantly reduced rate. A comparison of the mRNA expression profiles of SSEA-4^+^ cells and SSEA-4^−^ cells freshly isolated from the W0823-derived P2 xenografts showed that 1491 of a total of 54614 probe-sets (2.7%) were differentially expressed (fold changes > 1.5, *P* < 0.05) (also see the database deposit). Interestingly, in accordance with the assumption that SSEA-4^+^ osteosarcoma cells differentiate to produce SSEA-4^−^ cells, analyses of pathways revealed significant upregulation of several mesenchymal differentiation signature genes, such as *RUNX2*, *BMPR2*, *COL3A1*, *IBSP* and *ADIPOQ*, in SSEA-4^−^ cells (Fig. 3a and Supplementary Fig. 3a)^33^,^34^. Especially, Western blotting showed that freshly-isolated SSEA-4^−^ cells expressed elevated levels of the osteogenic transcription factor RUNX2 (Fig. 3b), which is indicative of osteoblastic commitment from MSCs or osteosarcoma TICs^16^. Additionally, the immunofluorescent inspection of Well5- or L1031-derived xenografts showed that some SSEA-4^−^ cells also expressed osteocalcin (OCN^hi^), a late-stage maturation marker of osteoblasts, but no SSEA-4^+^ cells did (Fig. 3c).

Next, we examined whether the induced mesenchymal differentiation of SSEA-4^+^ TICs *in vitro* would reduce tumorigenicity. As expected, the SSEA-4^+^ cell frequency declined when MG63 cells, U-2OS cells or Well5 cells were exposed to osteogenic or adipocytic differentiation inducers (Fig. 3d and Supplementary Fig. 3b) before any obvious apoptosis-induction was detectable (Supplementary Fig. 3c). As assayed by xenografting experiments, the tumorigenicity within the live Well5 cells was significantly decreased in conjunction with this process (Fig. 3e). Analogous to this, the frequency of SSEA-4^+^ cells declined along with the elongated post-confluence cultures in Well5 and MG63 cells (Fig. 3f), which, as previously reported^35^, induced osteogenic or adipocytic differentiation in osteosarcoma cells (Supplementary Fig. 3d). Again, a parallel gradual reduction in xenografting-TIC frequency was observed for Well5 cells (Fig. 3g and Supplementary Fig. 3e). Taken together, these results indicate that tumorigenicity was inversely associated with the differentiation status of osteosarcoma cells.

### mTORC1 activity maintains the immaturity of SSEA-4^+^ TICs

**Figure 4:**
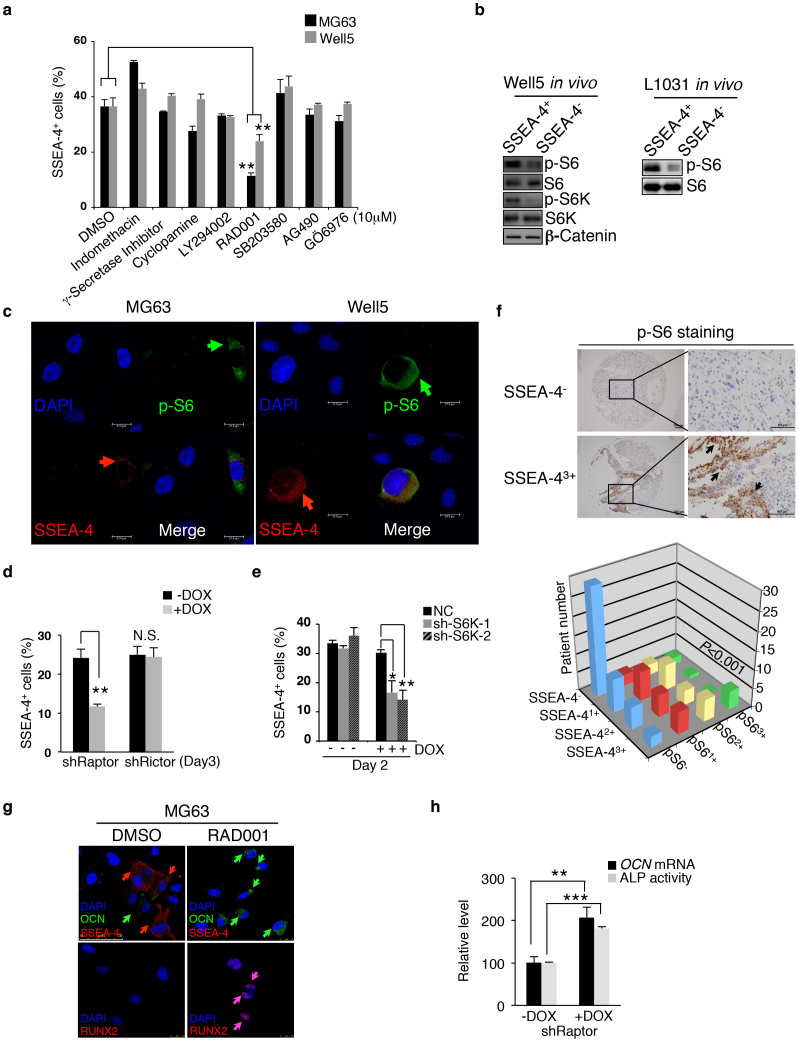
mTORC1 Activity Maintains SSEA-4^+^ TIC Frequency. (a) MG63 or Well5 cells were treated with negative control DMSO, Wnt-β catenin inhibitor indomethacin, Notch inhibitor γ-secretase inhibitor, Hedgehog inhibitor cyclopamine, PI3K-AKT inhibitor LY294002, mTOR inhibitor RAD001, p38 MAPK inhibitor SB203580, JAK-STAT inhibitor AG490, or PKC inhibitor GO6976 for 24 hours and the frequency of SSEA-4^+^ cells was measured by flow cytometry. Results are expressed as the mean ± SD (**P < 0.01). (b) Western blotting assays for phosphorylated levels of mTORC1 pathway components S6K or/and S6 as well as the β-catenin level in SSEA-4^+^ or SSEA-4^−^ cells freshly sorted from osteosarcoma xenografts. The cropped blots were run under the same experimental conditions. The full-length blots can be found in Supplementary Figure 8. (c) Immunofluorescent co-staining of SSEA-4 and p-S6 in cytospun MG63 and Well5 cells. (d–e) The effects of Dox-inducible Raptor or Rictor knockdown (d) or S6K knockdown (e) on the frequency of SSEA-4^+^ cells in MG63 cell culture. Results are expressed as mean ± SD (*P < 0.05, **P < 0.01). NC: control shRNA; shRaptor: shRNA for Raptor; shRictor: shRNA for Rictor; sh-S6K-1 and sh-S6K-2: shRNAs for S6K. (f) p-S6 level is positively correlated with SSEA-4 staining intensity among 98 human osteosarcoma samples (*P* = 0.000155). Representative immunohistochemical staining of p-S6 in one SSEA-4^−^ and SSEA-4^3+^ sample is shown on the left panel. Scale bars represent 100 μm. (g) MG63 cells were treated with DMSO or RAD001 for 3 days, then stained with the fluorescent antibodies against SSEA-4, OCN and RUNX2, and viewed microscopically. (h) ALP activity and *OCN* mRNA levels were measured in MG63 cells with or without Raptor knockdown (**P < 0.01, ***P < 0.001).

We next explored which molecular mechanisms maintain the immaturity of SSEA-4^+^ TICs. Actually, the pathway enrichment analysis described above (Fig. 3a) revealed that the functional states of multiple signal transduction and/or transcription pathways, including the Jak-Stat, the Wnt-β catenin, Notch and AKT-mTOR axes, exhibited significant differences when comparing SSEA-4^+^ to SSEA-4^−^ osteosarcoma cells (Supplementary Fig. 4a,b). To screen for the most critical one, we applied individual pathway inhibitors to ordinary cultures of Well5 and MG63 cells. The results showed that the mTOR inhibitor RAD001 but not others (including Jak, β-catenin and Notch inhibitors) quickly reduced the SSEA-4^+^ cell frequency (Fig. 4a). A detailed analysis of dose- or time-dependent inhibition of SSEA-4^+^ cell frequency by RAD001 or LY294002 indicated that the SSEA-4^+^ cell decrease was consistently associated with a reduction in mTORC1 (as indicated by p-S6K or p-S6 level) but not mTORC2 activity (as indicated by p-AKT^S473^ level) (Supplementary Fig. 4c,d)^36^. Consistent with this, mTORC1 activity, as indicated by the p-S6K or p-S6 level, was higher in SSEA-4^+^ cells than in SSEA-4^−^ cells asfreshly isolated from Well5- or L1031-derived xenografts, whereas there was no difference in the level of β-catenin (Fig. 4b). Moreover, the expression of SSEA-4 and p-S6 signals overlapped to a notable extent at the single-cell level (Fig. 4c), indicating that mTORC1 regulates SSEA-4^+^ TICs autonomously. To further examine whether mTORC2 was involved at all in this process^36^, we knocked down the Raptor, Rictor or S6K in MG63 cells individually using a Dox-inducible shRNA expression system (Supplementary Fig. 4e). Interestingly, the knockdown of mTORC1-related Raptor or S6K but not of mTORC2-related Rictor significantly decreased the frequency of SSEA-4^+^ cells (Fig. 4d, e and Supplementary Fig. 4f, g). Notably, in line with a previous study indicating that p-S6K levels in human osteosarcomas reliably predicted the prognosis^22^, the staining intensity of p-S6 and that of SSEA-4 were found to be positively correlated among 98 primary osteosarcoma specimens examined (Fig. 4f). This suggested a general contribution of elevated mTORC1 activity to the maintenance of SSEA-4^+^ TICs in human osteosarcoma cells.

As expected, mTORC1 inactivation by RAD001 drove MG63 and Well5 SSEA-4^+^ cells into osteogenic differentiation as evident by the upregulation of RUNX2 and OCN (Fig. 4g). Surprisingly, some of these osteosarcoma cells actually reached the terminal osteocytic stage as evident by positive Alizarin Red S staining (Supplementary Fig. 4h). Likewise, Raptor knockdown but not Rictor knockdown resulted in the upregulation of both *OCN* mRNA and ALP–another osteogenic maturation marker (Fig. 4h and Supplementary Fig. 4i). Nevertheless, the upregulation of RUNX2 (Supplementary Fig. 4g) but not of *OCN* mRNA levels or ALP activity could be caused by S6K knockdown (Supplementary Fig. 4j), thus indicating that SSEA-4^−^ osteosarcoma cells are a heterogeneous population lying at discrete differentiation stages, and that S6K inhibition alone promoted only an osteogenic commitment of SSEA-4^+^ TICs but not further maturation.

### Partially differentiated SSEA-4^− ^osteosarcoma cells dedifferentiate to regenerate SSEA-4^+^ TICs

**Figure 5:**
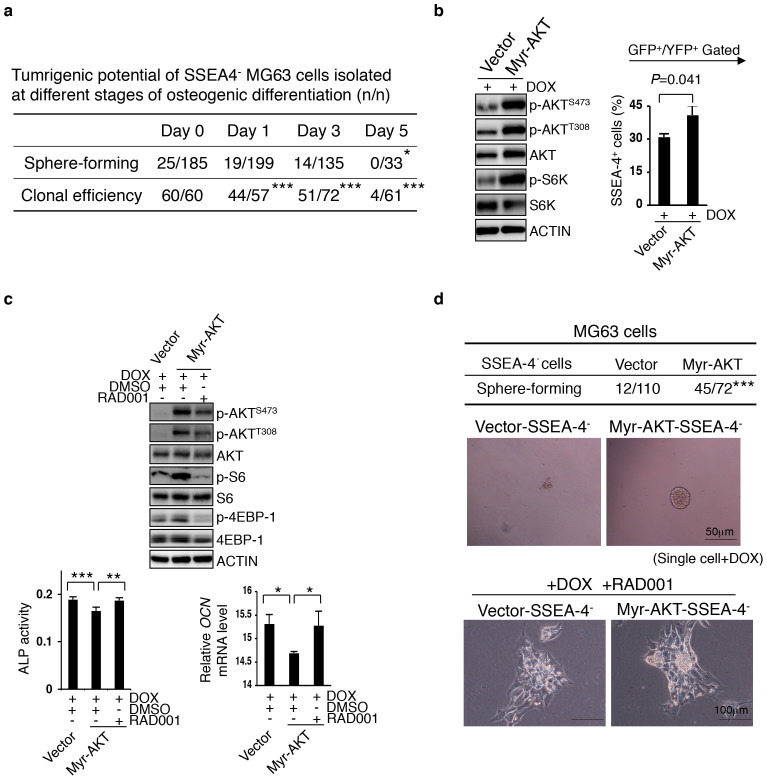
mTORC1 Supports the De-differentiate Potential of Early SSEA-4^−^ Progeny. (a) SSEA-4^−^ cells were sorted at different time points during MG63 cells' differentiation to osteocytes and individually inoculated into fresh medium for measuring their clonal efficiency or into special medium for measuring their tumorsphere-forming potential (*P < 0.05, ***P < 0.001). (b) Enforced activation of AKT-mTOR pathway increases SSEA-4^+^ cell frequency in MG63 cells undergoing mesenchymal differentiation. Transduced cells were plated at 60% confluence and the expression of Myr-AKT was induced by Dox; SSEA-4^+^ cell frequency was measured 48 hours later. Results are shown as means ± SDs. The cropped blots were run under the same experimental conditions. The full-length blots can be seen in Supplementary Figure 8. (c) mTOR inhibitor relieves the AKT activation-caused differentiation arrest of MG63 cells. Data for ALP activity and *OCN* mRNA levels are presented as means ± SDs (*P < 0.05, **P < 0.01, ***P < 0.001). The cropped blots were run under the same experimental conditions. The full-length blots can be seen in the Supplementary Figure 8. (d) Tumorsphere-forming rates of single SSEA-4^−^ MG63 cells expressing vector or Myr-AKT (as in b or c) are shown on the upper panel (***P < 0.001). The enhancing effect of Myr-AKT induction on the tumorsphere-forming potential of SSEA-4^−^ cells was abolished by RAD001 (bottom panel).

Notably, a partial retention of tumorigenicity by SSEA-4^−^ osteosarcoma cells was actually detectable by xenografting or tumorsphere-forming assay (Fig. 1d–f). Most of these cells were still highly immature osteosarcoma cells as marked by Oct3/4 expression (Fig. 2b, c). As suggested by recent studies in osteosarcoma and other types of aggressive tumors^37^,^38^,^39^, we suspected that a dedifferentiation potential existed within the progeny of SSEA-4^+^ TICs, which contributed to the retention of tumorigenicity. To test this, we retrieved single SSEA-4^−^ MG63 or Well5 cells from the ordinary passage (cellular confluence was <80%) and inoculated them into 96-well plates with nutrient-replenished medium. Indeed, the colony-forming rate of SSEA-4^−^ cells was comparable to that of SSEA-4^+^ cells, although the formation of colonies by SSEA-4^−^ cells usually took longer times (≈21 days versus 10 days) (Supplementary Fig. 5a), thus excluding the possibility that colony formation by SSEA-4^−^ cells was the result of contamination by SSEA-4^+^ cells. Interestingly, SSEA-4^+^ cells were consistently detected within colonies generated by SSEA-4^−^ cells (Supplementary Fig. 5b), indicating that the dedifferentiation of SSEA-4^−^ cells explained their colony-forming ability. Nevertheless, it was interesting to note that when mesenchymal differentiation inducers were added to the culture, the clonal recovery rate and the tumorsphere-forming rate among live SSEA-4^−^ cells progressively decreased along with induced osteogenic differentiation (Fig. 5a), suggesting that dedifferentiation potential was inversely correlated to the maturation status of heterogeneous SSEA-4^−^ osteosarcoma cells.

### mTORC1 induces a bias toward dedifferentiation rather than terminal differentiation in SSEA-4^−^ osteosarcoma cells

In parallel to this flexible tumorigenicity observed in osteosarcoma cells, it is well known that the mTORC1 activity is reversibly modulated by the AKT and AMPK pathways^40^,^41^. As expected, Dox-induced expression of Myr-AKT activated mTORC1, which in turn prevented the loss of the SSEA-4^+^ cells during the elongated period of culturing (Fig. 5b). Parallel measurements of ALP and *OCN* indicated that the induced terminal differentiation of MG63 cells was partially arrested (Fig. 5c), a result that was then reversed by RAD001, which preferentially targeted the mTORC1 complex (Fig. 5c). Conversely, glucose depletion for 5 days, which is known to inactivate mTORC1 via AMPK activation, induced terminal osteoblastic differentiation in MG63 cells (Supplementary Fig. 5c, d), and accordingly decreased SSEA-4^+^ cell frequency and tumorsphere-forming rate in these cells (Supplementary Fig. 5e, f). Notably, an inducible Myr-AKT activity endowed SSEA-4^−^ cells with a measurable gain in tumorsphere-forming potential, which was abolished by RAD001 (Fig. 5d). Taken together, these findings indicate that mTORC1 maintains the SSEA-4^+^ TIC pool not only by supporting their self-renewal but also by biasing the dedifferentiation of certain SSEA-4^−^ progeny at the expense of their terminal maturation (Supplementary Fig. 5g). In line with this, *in vitro* pretreatment with RAD001 significantly decreased the tumorigenic potential of MG63 cells and Well5 cells (Supplementary Fig. 5h, i), which was mimicked by Dox-induced Raptor knockdown but not by Rictor knockdown after transient treatment (Supplementary Fig. 5j).

### mTOR inactivation causes the terminal differentiation of apical SSEA-4^+^ TICs *in vivo*

**Figure 6:**
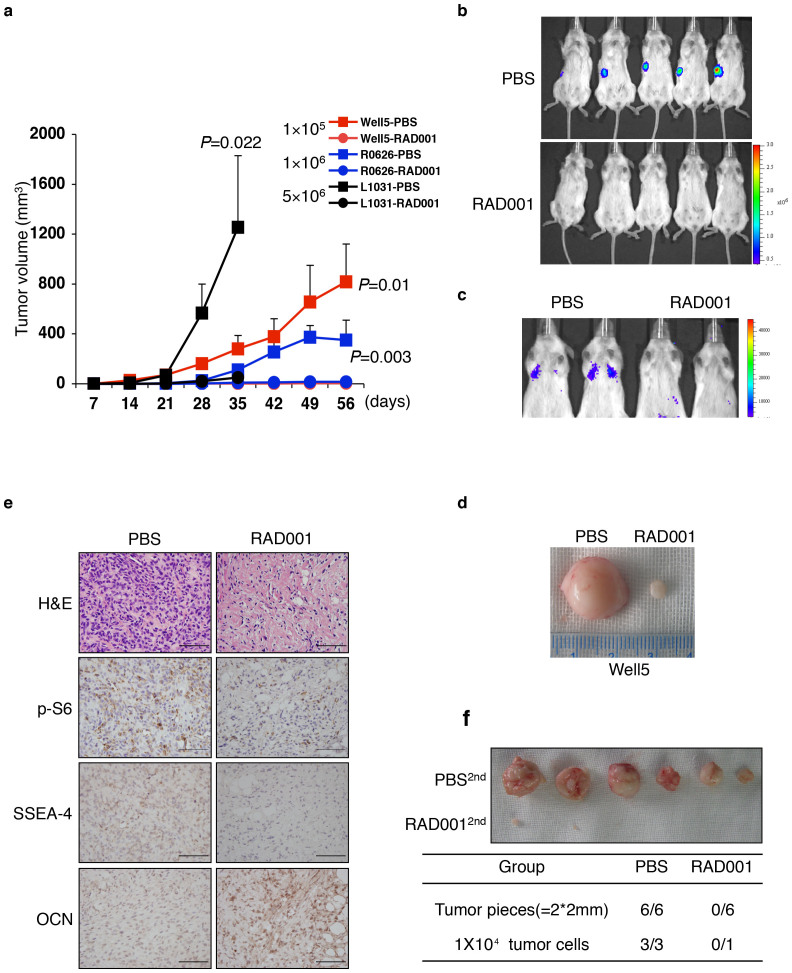
Terminal Differentiation Induction by mTOR-inactivation Decreases SSEA-4^+^ TICs *in vivo*. (a) SSEA-4^+^ cells (1–50 × 10^5^) from the different resources as indicated were subcutaneously inoculated into NOD/SCID mice. The oral administration of PBS (filled box) or 5 mg/kg RAD001 (filled circle) commenced 2 days later. Tumor volumes are shown as the means ± SDs, n = 3–5. (b–c) Representative images of whole-body (b) or lung metastasis (c) bioluminescence 4 weeks following subcutaneous (b) or tail vein injection (c) of 1 × 10^5^ PBS- or RAD001-treated SSEA-4^+^ Well5 cells (as in (a)) into NOD/SCID mice. (d) Two representative tissue samples retrieved from the PBS-treated and RAD001-treated groups, respectively. (e) Immunohistochemical staining of p-S6, SSEA-4, or OCN in xenografts retrieved from the PBS-or RAD001-treated group, as in (a and b). Scale bars represent 100 μm. HE: hematoxylin-eosin. (f) Secondary tumorigenic xenograft formation of tumor tissue or cells after post-PBS or -RAD001 treatment, as in (a and b) (upper panel). Secondary tumorigenic xenografting rates (n/n) are summarized in the bottom table (*P* < 0.01).

mTORC1 activity is elevated by p53 deficiency through attenuation of AMPK activity and potentially by various types of other osteosarcoma-related oncogenic events^40^,^42^,^43^,^44^,^45^. Nevertheless, we argued that mTORC1 activity is also subject to negative regulation by various types of environmental cues, such as the lack of nutrients that significantly decreased mTORC1 activity by activating AMPK in p53 or/and Rb-deficient osteosarcoma cells (Supplementary Fig. 5c–f). Since previous studies using mTOR inhibitors showed differential inhibitory effects on the *in vivo* growth of different osteosarcoma cell lines^23^,^24^,^25^,^26^, we examined whether mTOR inactivation alone would cause loss of tumorigenicity in SSEA-4^+^ TICs and their progeny by enforcing terminal differentiation *in vivo*. Interestingly, following the subcutaneous or intravenous inoculation of NOD/SCID mice with 1–50 × 10^5^ SSEA-4^+^ osteosarcoma cells freshly- isolated from 3 different xenografts, RAD001 gavage for about 4–8 weeks significantly inhibited the formation and/or growth of subcutaneous or lung xenografts in these animals (Fig. 6a–c). Notably, pathological studies showed that compared to bulky PBS-treated tumorigenic xenografts, the tiny residual xenografts recovered from the RAD001-treated group were mostly the accumulation of a cellular collagenous masses akin to osteoid structures. These masses were only sparsely infiltrated by SSEA-4^−^ p-S6^low^ OCN^hi^ mature osteogenic cells (Fig. 6d, e). To test whether tumorigenicity was decreased by RAD001 treatment, we then re-inoculated the retrieved xenograft tissues or isolated tumor cells into secondary recipients without further intervention. Strikingly, no tumorigenic engraftments were detected after 7 inoculations of RAD001-treated xenografts, whereas all 9 comparable inoculations from PBS-treated xenografts generated the palpable tumors (Fig. 6f). This demonstrates a strong inhibitory effect of mTOR inactivation on the propagation of SSEA-4^+^ TICs through the enforcement of terminal maturation. On the other hand, although systemic chemotherapy with Methotrexate (MTX) also partially inhibited the growth of xenografts, abundant SSEA-4^+^ p-S6^+^OCN^−^ immature TICs were detected (Supplementary Fig. 6a, b), and these cells generated the tumorigenic xenografts upon the secondary inoculation (data not shown).

### mTORC1 inactivation derepresses p27 resulting in terminal differentiation of p53/Rb-deficient osteosarcoma cells

As S6K inactivation promoted only a partial osteogenic differentiation of SSEA-4^+^ TICs (Supplementary Fig. 4j), we wanted to investigate the downstream mechanisms of mTORC1 in the regulation of dedifferentiation versus terminal maturation of partially differentiated SSEA-4^−^ osteosarcoma cells, as this marks a critical point for retention or elimination of tumorigenicity. In this regard, previous studies indicated that cell-cycle exit or reentry controlled by p27 level/activity is a prerequisite for the terminal differentiation or malignant transformation of osteoblasts, respectively^15^,^16^. Especially, p27 levels in high-grade human osteosarcoma were found to be greatly reduced compared to such levels in low-grade osteosarcoma^16^. Interestingly, among different osteogenic differentiation models tested, we noticed a common molecular occurrence: a reduction in mTORC1 activity was accompanied selectively by a progressive upregulation of the cell cycle inhibitor p27 (Supplementary Fig. 5c, Supplementary Fig. 7a, b and data not shown), while neither p53 nor Rb expression was upregulated in Rb-null Well5 cells nor in p53-null MG63 cells during induced mesenchymal differentiation (Supplementary Fig. 7c). Indeed, p27 levels in SSEA-4^+^ TICs-initiated xenografts were significantly upregulated by RAD001 (Supplementary Fig. 7d). In line with this, previously studies have demonstrated that p27 repression represents a key mechanism whereby mTOR activity supports cellular proliferation^46^,^47^,^48^. We confirmed that RAD001 elevated the levels of *p27* mRNA in parallel with *OCN* mRNA levels (Supplementary Fig. 7e).

**Figure 7:**
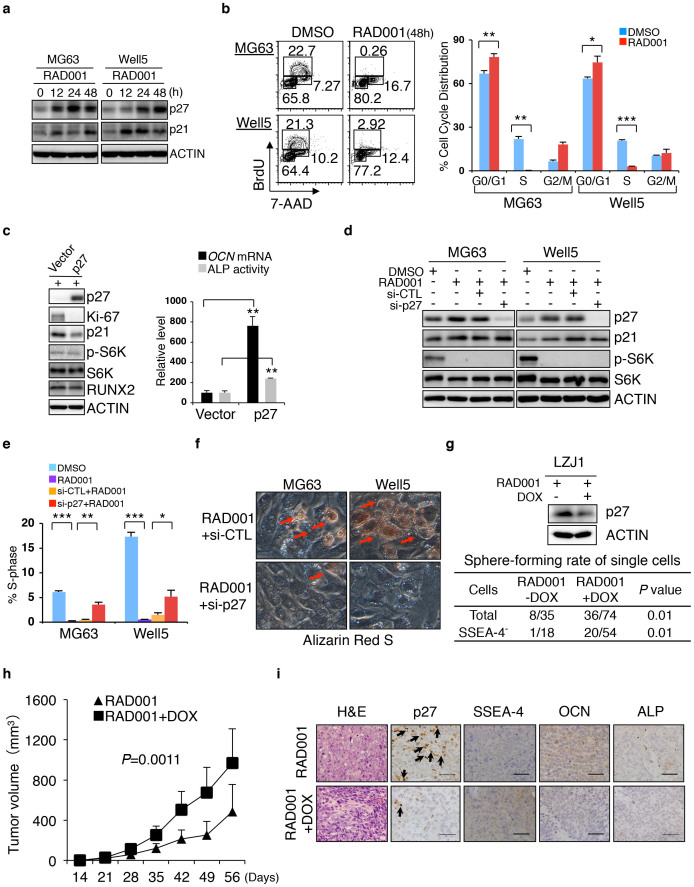
p27-initiated Cell-cycle Exit Contributes to mTOR Inactivation-induced Terminal Osteogenic Differentiation of SSEA-4^+^ TICs. (a) Western blotting assays for p27 and p21 in MG63 and Well5 cells treated with DMSO or RAD001 for varying lengths of time. The cropped blots were run under the same experimental conditions. The full-length blots can be viewed in Supplementary Figure 8. (b) Cell cycle status of RAD001-treated MG63 and Well5 cells. Data are presented as means ± SDs (*P < 0.05, **P < 0.01, ***P < 0.001). (c) Inducible expression of exogenous p27 elevates ALP activity and *OCN* mRNA levels in MG63 cells. Left panel, Western blot assay of p27-overexpressing MG63 cells. Data are presented as the means ± SDs (**P < 0.01). (d) Western blot assay on RAD001-treated MG63 and Well5 cells with or without p27 knockdown. si-CTL: control siRNA; si-p27: p27 siRNA mixture. The cropped blots were run under the same experimental conditions. The full-length blots can be viewed in Supplementary Figure 8. (e–f) S-phase distribution (e) and terminal osteogenic maturation (f) of RAD001-treated MG63 and Well5 cells with or without p27 knockdown were evaluated by flow cytometry and Alizarin Red-S staining, respectively. (g) Dox-induced p27 knockdown enhanced the tumorsphere-forming ability of LZJ1-derived total or SSEA-4^−^ osteosarcoma cells. (h) Monitoring of the *in vivo* growth of LZJ1-derived osteosarcoma cells which were treated with either RAD001 or RAD001 plus p27 knockdown. (i) Immunohistochemical staining of p27, SSEA-4, OCN or ALP in xenografts recovered from RAD001 or RAD001 plus p27 knockdown groups. Scale bars represent 100 μm. HE: hematoxylin-eosin.

Indeed, consistent with a progressive elevation in p27 but not in p21 levels (Fig. 7a and supplementary Fig. 7f), RAD001 induced a pre-S phase cell cycle arrest in MG63, U-2OS and Well5 cells prior to inducing apoptosis (Fig. 7b and Supplementary Fig. 7g–h). These results indicated that, analogous to a well-documented role for p21 upregulation in driving the differentiation of certain types of osteosarcoma cell lines^49^, p27 upregulation represents a critical molecular event following mTOR inactivation in SSEA-4^+^ TIC-derived progeny, especially for their late-stage differentiation. Consistent with this observation, while Raptor knockdown upregulated both p27 and p21, Rictor knockdown upregulated p21 but not p27 in p53-deficient MG63 cells (Supplementary Fig. 7i). Knockdown of Raptor resulted in obvious cell-cycle arrest (Supplementary Fig. 7j), while such arrest was not seen in Rictor knockdown.

In MG63 cells, *p27* induction alone enforced osteogenic differentiation, probably by facilitating Runx2 activity^16^, (Fig. 7c); such induction also inhibited the dedifferentiation of SSEA-4^−^ cells (Supplementary Fig. 7k) even in the presence of a reduction in p21, which was most likely compensatory (Fig. 7c). Conversely, *p27* knockdown, while causing no obvious alteration to p-S6K or p21 levels (Fig. 7d), alleviated cell cycle arrest and hindered the terminal osteogenic differentiation of both MG63 and Well5 cells treated with RAD001 *in vitro* (Fig. 7e,f). Accordingly, the tumorsphere-forming potential of RAD001-pretreated total or SSEA-4^−^ L1031 osteosarcoma cells was significantly restored by *p27* knockdown (Fig. 7g). As expected, the induced knockdown of p27 significantly alleviated the growth inhibitory effect of RAD001 on LZJ1-derived xenografts (Fig. 7h) in which SSEA-4^+^, ALP^low^ or OCN^low^ immature osteosarcoma cells were abundantly retained (Fig. 7i).

## Discussion

A previous study demonstrated that the Oct3/4 promoter is active in certain types of osteosarcoma TICs^50^, while the reason for the obvious discrepancy in the prognoses of clinical cases was not addressed. In this study, we show that SSEA-4^+^ cells represent a particular subset of TICs in human osteosarcomas. These SSEA-4^+^ cells represent a small fraction of Oct3/4^+^ tumor cells, whose abundance is the hallmark of high-risk cases but not those with good prognosis following conventional therapy. The co-purification of SSEA-4 and Oct3/4 exclusively in high-grade cases supports the notion that a reprogramming process may accompany the creation of TICs for high-grade osteosarcoma cases but not for low-grade cases. During such reprogramming, cells at least partially approach the level of MSCs or even show certain ES cell-like features^30^,^32^,^51^.

Theoretically, factors associated with the prognosis of any type of cancer ought to be ones that play a critical role in the regulation of tumorigenesis. In this regard, it was previously observed that a reduced p27 level or an elevated level of p-S6K level was found in association with high-grade rather than low-grade osteosarcoma samples^16^,^22^. In accordance with this, multiple independent studies have already demonstrated that mTORC1 activity represses p27 expression in a variety of tumor types^46^,^47^,^48^. In this study, we demonstrated that SSEA-4^+^ TIC frequency is associated with mTORC1 activity to predict prognosis in osteosarcoma. Taken together, these observations suggest that a possible link exists between mTORC1 activity and p27 levels in the regulation of the SSEA-4^+^ TIC pool.

The question, then, is how mTORC1-p27 regulates the pool of SSEA-4^+^ TICs. Previous *in vitro* studies that used different cell models resulted in differing conclusions regarding the role of mTORC1 or/and mTORC2 in controlling stemness versus maturation of osteosarcoma cells^52^,^53^. Recent studies indicate that aggressive types of malignancies are characterized by tumor cells that possess dedifferentiation potential^37^,^38^. In the case of osteosarcoma, since the dedifferentiation of p53- or/and Rb-deficient mature osteoblasts underlies their malignant transformation^4^,^11^,^12^,^13^, it is not surprising to find that SSEA-4^−^ osteosarcoma cells retain the capacity to dedifferentiate, thus regenerating SSEA-4^+^ TICs.

There are redundant cell-cycle regulators that potentially drive osteoblasts out of the cell-cycle, which in turn hinders the dedifferentiation potential of osteosarcoma cells^15^,^16^. The genetic inactivation of the Rb pathway, including the deletion of CDK inhibitors p16^INK4A^ and the pocket protein Rb, as well as amplification of *CDK4*, is part of the reason why osteosarcoma cells are able to avoid permanent cell-cycle exit. The repression of *p21* expression, as implicated by the genetic inactivation of *p53* and *p14^ARF^* as well as the amplification of *MDM2* (p14^ARF^-MDM2-p53-p21) or the functional activation of *USP1-IDs*^49^, contributes to tumorigenesis by blocking osteosarcoma cells from cell-cycle exit. However, it seems that these abnormalities taken together still do not prevent the possible upregulation of *p27*, which has been shown to be sufficient to drive Rb-deficient osteoblasts out of the cell-cycle^54^. In this study, we show that mTORC1 not only supports SSEA-4^+^ TIC expansion via activating S6K, but also determines the retention of the tumorigenicity of p53/Rb-deficient osteosarcoma cells by repressing p27. Moreover, it is highly possible that p27 may also play an additional role in promoting the early or intermediate differentiation phase of SSEA-4^+^ TICs, for example, by facilitating Runx2 function through maintenance of residual Rb in its hypophosphorylated form, or other unknown pathways^16^.

It is interesting to consider what factors regulate the increases and decreases of mTORC1 activity in osteosarcoma cells growing *in vivo*. Previous work has indicated that the deficiency of Rb or p53 activities may increase mTORC1 activity in osteosarcoma cells via compromising AMPK activity or elevating mTOR expression^43^,^44^. Nevertheless, we argue that a variety of environmental cues as well as other osteosarcoma-related genetic mutations would influence mTOR activity^13^,^21^. We showed that the glucose-withdrawal activated AMPK to inhibit mTORC1 activity in p53-deficient MG63 cells. On the other hand, our data also support previous findings that growth factor-stimulated mTORC1 activation promotes TIC activity in osteosarcomas resulting in metastasis^40^. Nevertheless, our results do not exclude an additional contribution by the inactivation of mTORC2, probably secondary to mTORC1 inactivation^55^ in the restriction of tumorigenicity in high-grade osteosarcoma^26^. When considered in their broader context, our observations indicate that mTOR-targeting represents a promising way to improve the treatment of high-grade osteosarcoma, especially those characterized by the presence of highly immature SSEA-4^+^ TICs.

## Methods

### Patients

All of the patients included in this study were admitted to Shanghai Rui-Jin Hospital during the last 12 years. All patients received systemic chemotherapy according to the IOR/OS-N4 protocol, plus surgery^18^. All patients provided written informed consent for the use of their tumor samples under a protocol approved by the ethics committee of Shanghai Rui-Jin Hospital.

### Human Osteosarcoma Xeno-transplantation

Human primary osteosarcoma samples were obtained in accordance with the ethical guidelines established by Shanghai Rui-Jin Hospital. Fresh osteosarcoma samples were dissected into small pieces (<3 × 3 × 1.5 mm^3^) within an hour after surgery. The pieces were then implanted subcutaneously into the two flanks of 6 to 8 week-old female NOD/SCID mice through a 3-mm incision, which was then wrapped twice with a 5–0 suture. All animal experiments were carried out in accordance with the approved guidelines provided by the Laboratory Animal Resource Center of Shanghai Jiao Tong University School of Medicine.

For the isolation and further passage of osteosarcoma cells from the xenografts, tumor tissues were dissected into small pieces (2 × 2 × 1 mm^3^) and then incubated with Collagenase Type IV (Invitrogen) in IMDM medium (250 units/ml) at 37°C for 1 hour. Single cell suspensions were obtained by filtering the collagenase-treated samples through a 70-μm nylon cell strainer (BD Falcon, Bedford, USA). One thousand to 1 million unsorted, sorted SSEA-4^+^ or SSEA-4^−^ osteosarcoma cells suspended in PBS were then injected into the distal ends of femurs or subcutaneously into the flanks of 6 to 8 week-old female NOD/SCID mice. Tumor volumes were estimated by following an established formula (tumor volume = π/6 × 0.5 × length × width^2^).

### Flow Cytometric Analysis and Cell Sorting

Cells were suspended in FACS buffer (PBS containing 1% FBS, with or without 0.1% Na_3_N) and subsequently stained with fluorochrome-conjugated antibodies. Flow cytometric data were collected using an LSRII flow cytometer (Becton Dickinson, Franklin Lakes, NJ, USA) or a MoFlo flow-sorter (Beckman coulter, Fullerton, CA, USA), and were analyzed with either FlowJo software (TreeStar, Ashland, OR, USA) or Summit software (Beckman coulter). Cells were sorted by MoFlo into DMEM supplemented with 2% FBS.

### Gene Expression Profiling

Total RNA was extracted from sorted SSEA-4^+^ and SSEA-4^−^ cells using Trizol (Invitrogen). Three biological replicates were performed and all samples were subjected to strict quality control. Gene expression profiling was conducted by Shanghai Biotechnology Corporation using Affymetrix U133 plus 2.0 arrays (Affymetrix, Santa Clara, CA). All data were analyzed according to the manufacturer's protocol. Raw data generated from Affymetrix CEL files were normalized by RMA background correction; values were log_2_ transformed. Comparison of the data sets by *t* test showed that 1491 of the total of 54614 probe-sets (2.7%) were differentially expressed (fold changes > 1.5, *P* < 0.05). For the enrichment of *P* values of each GO term, we used Fisher's exact test to calculate *P* values and R package stats to calculate FDR (q value) by BH method (www.r-project.org). All microarray data have been deposited with Gene Expression Omnibus (GEO) under accession number GSE58209.

### Tissue Microarrays and Immunohistochemical Analyses

The osteosarcoma tissue microarray was provided by the Department of Pathology of Rui-Jin Hospital. Two representative cores of each paraffin-embedded tissue sample (1 mm in diameter) were selected for immunohistochemical staining, which was performed on 5 μm-thick paraffin-embedded sections. All experimental protocols for osteosarcoma samples were approved by the ethics committee of Shanghai Rui-Jin Hospital.

### Statistical Analysis

Data are presented as arithmetic means ± SD (standard deviations). Kaplan-Meier survival analysis, student's *t* tests or χ^2^ tests were used to calculate *P* values where appropriate. *P* < 0.05 was considered to be significant.

## Author Contributions

W.Z. and J.Z. designed the study. W.Z., M.-L.D. and J.-N.Z. performed the experiments and interpreted the data. W.-B.Z., Y.-H.S., J.-R.Q., X.-Y.D. and L.-F.D. provided the clinical samples or experimental material and were involved in interpreting data. J.Z. supervised the entire project and wrote the manuscript.

## Supplementary Material

Supplementary Information Supplementary information
