# Author Correction: mTORC1 Maintains the Tumorigenicity of SSEA-4 ^+^ High-Grade Osteosarcoma

**DOI:** 10.1038/s41598-021-00930-2

**Published:** 2021-10-27

**Authors:** Wu Zhang, Meng-Lei Ding, Jia-Nian Zhang, Jian-Ru Qiu, Yu-Hui Shen, Xiao-Yi Ding, Lian-Fu Deng, Wei-Bin Zhang, Jiang Zhu

**Affiliations:** 1 State Key Laboratory for Medical Genomics, Shanghai Institute of Hematology and Collaborative Innovation Center of Hematology, Rui-Jin Hospital Affiliated to Shanghai Jiao-Tong University School of Medicine, Shanghai, 200025 People’s Republic of China; 2 Division of Orthopedics, Shanghai Institute of Traumatology and Orthopaedics, Shanghai, 200025 People’s Republic of China; 3 Shanghai Institute of Digestive Surgery, Shanghai, 200025 People’s Republic of China; 4 Department of Radiology, Rui-Jin Hospital, Shanghai, 200025 People’s Republic of China; 5 Collaborative Innovation Center of Systems Biomedicine, Shanghai, 200025 People’s Republic of China

Correction to: *Scientific Reports* 10.1038/srep09604, published online 08 April 2015

This Article contains an error in Figure 6, where the incorrect image was used for Figure 6c. The correct Figure 6 and its accompanying legend appear below.

**Figure 6:**
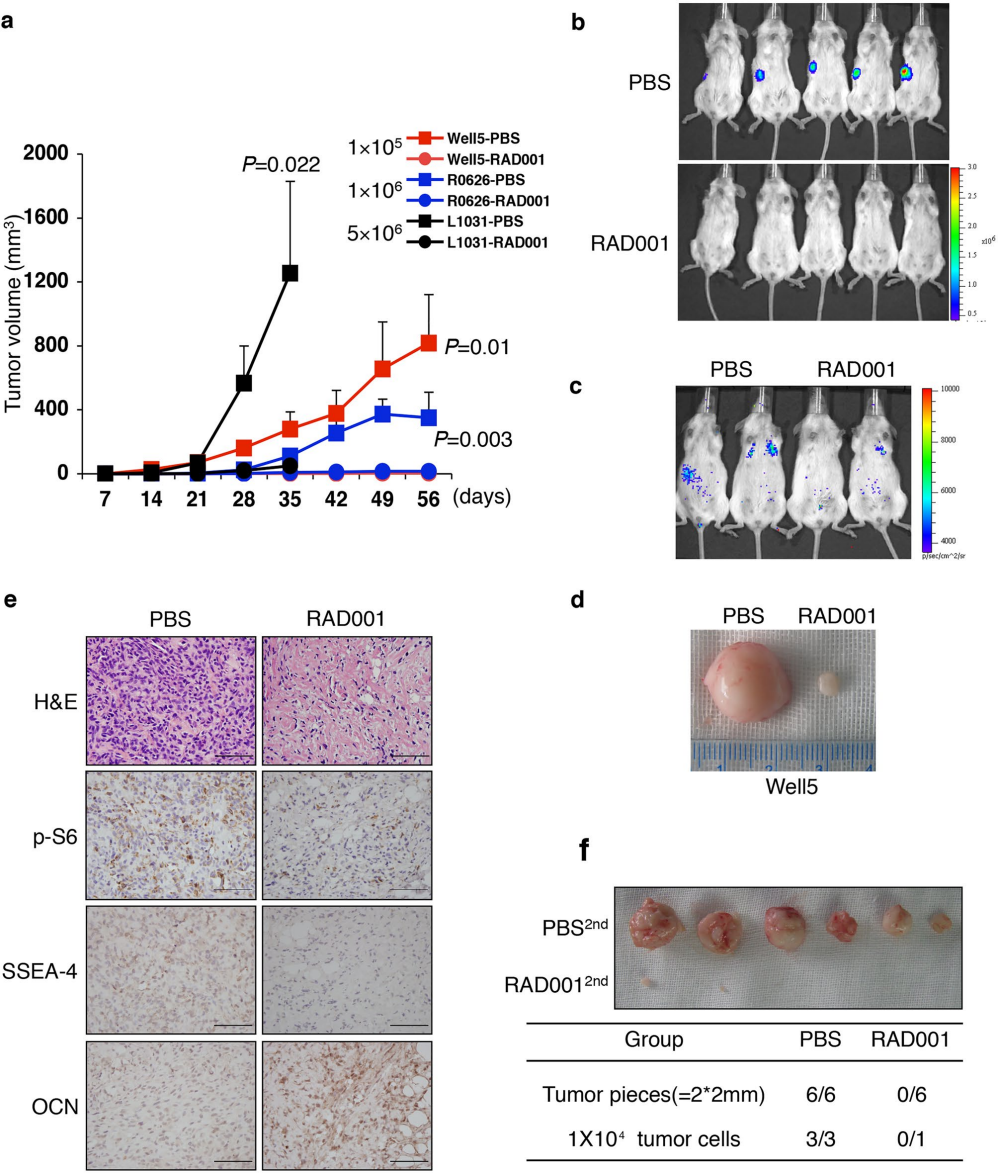
Terminal differentiation induction by mTOR-inactivation decreases SSEA-4^+^ TICs in vivo. (**a**) SSEA-4^+^ cells (1–50 × 10^5^) from the different resources as indicated were subcutaneously inoculated into NOD/SCID mice. The oral administration of PBS (filled box) or 5 mg/kg RAD001 (filled circle) commenced 2 days later. Tumor volumes are shown as the means ± SDs, n = 3–5. (**b**, **c**) Representative images of whole-body (**b**) or lung metastasis (**c**) bioluminescence 4 weeks following subcutaneous (**b**) or tail vein injection (**c**) of 1 × 10^5^ PBS- or RAD001-treated SSEA-4^+^ Well5 cells (as in (**a**)) into NOD/SCID mice. (**d**) Two representative tissue samples retrieved from the PBS-treated and RAD001-treated groups, respectively. (**e**) Immunohistochemical staining of p-S6, SSEA-4, or OCN in xenografts retrieved from the PBS-or RAD001-treated group, as in (**a**, **b**). Scale bars represent 100 μm. HE: hematoxylin–eosin. (**f**) Secondary tumorigenic xenograft formation of tumor tissue or cells after post-PBS or -RAD001 treatment, as in (**a**, **b**) (upper panel). Secondary tumorigenic xenografting rates (n/n) are summarized in the bottom table (*P* < 0.01).

