# Supplementary material for: mTORC1 Maintains the Tumorigenicity of SSEA-4+ High-Grade Osteosarcoma
Source: Sci Rep. 2015 Apr 8;5:9604. doi: 10.1038/srep09604 (PMC4389812; doi:10.1038/srep09604)
Supplement: Supplementary Information [file srep09604-s1.doc]

**Supplementary information**

**mTORC1 Maintains the Tumorigenicity of SSEA-4^+^ High-grade Osteosarcoma**

Wu Zhang^1, 5^*, Meng-Lei Ding^1^*, Jia-Nian Zhang^3^, Jian-Ru Qiu^2^, Yu-Hui Shen^2^, Xiao-Yi Ding^4^, Lian-Fu Deng^2^, Wei-Bin Zhang^2^, and Jiang Zhu^1, 5^

^1^State Key Laboratory for Medical Genomics, Shanghai Institute of Hematology and Collaborative Innovation Center of Hematology,

^2^Division of Orthopedics and Shanghai Institute of Traumatology and Orthopaedics,

^3^Shanghai Institute of Digestive Surgery,

^4^Department of Radiology,

Rui-Jin Hospital,

^5^Collaborative Innovation Center of Systems Biomedicine,

Shanghai 200025; People’s Republic of China.

*These authors contributed equally to this work.

Correspondence should be addressed to Jiang Zhu ([zhujiang@shsmu.edu.cn](mailto:zhujiang@shsmu.edu.cn)) or Wei-Bin Zhang ([weibin@medmail.com.cn](mailto:weibin@medmail.com.cn)).

**
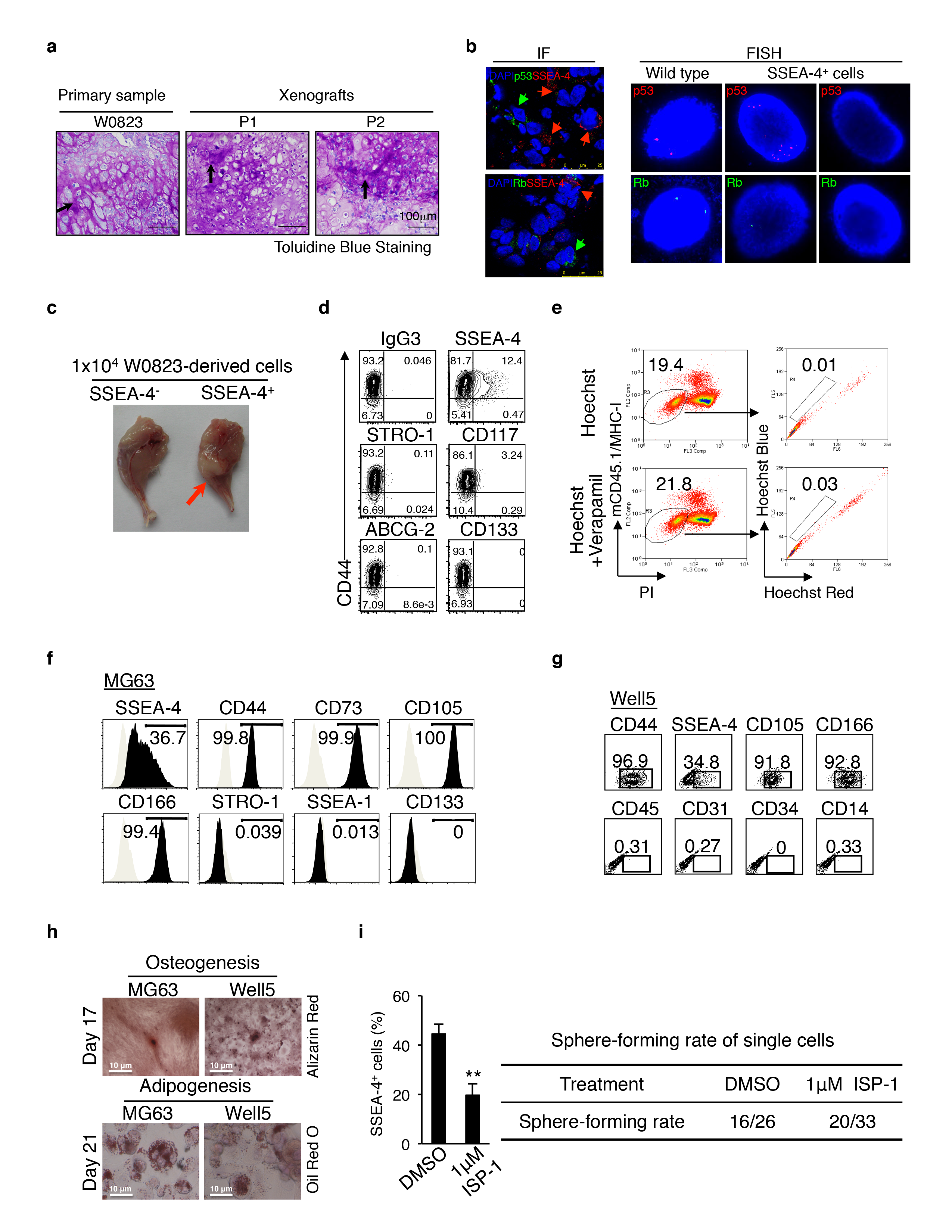
**

**Supplementary Figure 1.**  **The Biological Characterization of Osteosarcoma TICs.**

(a) Toluidine blue staining of primary osteosarcoma sample W0823 and its P1 and P2 xenografts for the detection of the osteoid structures, indicated by the black arrows. (b) Immunofluorescent staining of frozen sections of primary osteosarcoma specimens for SSEA-4, Rb, and p53. Representative FISH for *p53* and *Rb* abnormalities in sorted SSEA-4^+^ cells from primary osteosarcoma specimens. (c) Orthotopic inoculation of 1×10^4^ SSEA-4^+^ but not SSEA-4^-^ W0823-derived cells produced tumorigenic xenografts. (d) Human CD44-positive xenograft cells were co-stained for SSEA-4 or several other mesenchymal/osteogenic markers and analyzed by flow cytometry. (e) Measurement of SP (side population) cells within the W0823-derived P2 xenograft cells is shown. Prior to SP cell analysis, apoptotic and mouse cells had been gated out by PI and mCD45/MHC-I stainings. (f) Flow cytometric analysis of the antigenic expression profile of human osteosarcoma cell line MG63 cells. (g) Immunophenotypic profiling of Well5 cells by flow cytometry. Well5 was the osteosarcoma cell line derived from the *in vitro* passage of primary sample W0823. Well5 cells were cultivated at 5% CO_2_, 37^o^C in DMEM supplemented with L-glutamine, penicillin/streptomycin and 10% fetal bovine serum. (h) The osteogenic and adipogenic differentiation potentials of Well5 cells, in parallel to those of MG63 cells, were measured by Alizarin Red staining, which is indicative of mature osteogenic cells and Oil Red-O staining, which is indicative of mature adipogenesis. (i) MG63 cells were treated with DMSO or the SSEA-4 synthesis inhibitor ISP-1 for 5 days. The frequency of SSEA-4^+^ cells was then measured by flow cytometry (left panel). The tumorsphere-forming rates of MG63 cells post-treatments are listed in the right table (*P* = 0.94).

**
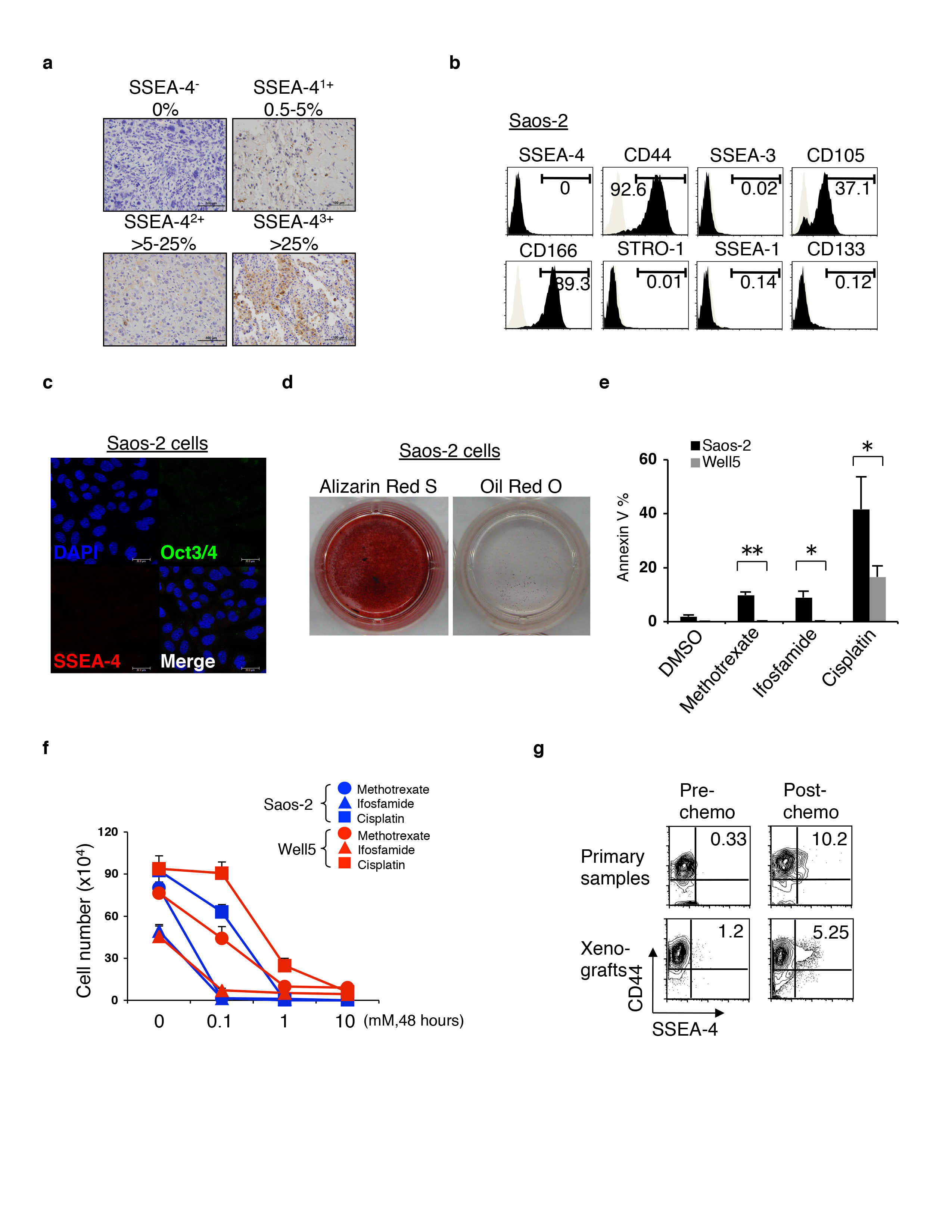
**

**Supplementary Figure 2.**  **SSEA-4^+^ Osteosarcoma and SSEA-4^-^ Osteosarcoma Are Two Distinct Subtypes of Malignancy.**

(a) SSEA-4 staining patterns representative of four grades of human primary osteosarcoma samples: SSEA-4^-^, SSEA-4^1+^, SSEA-4^2+^ and SSEA-4^3+^. (b) Flow cytometric analysis of the antigen expression profile of human osteosarcoma Saos-2 cells. (c) Cytospun Saos-2 cells were co-stained with fluorescent antibodies against Oct3/4 or SSEA-4. (d) The osteogenic and adipogenic differentiation potentials of Saos-2 cells were measured by Alizarin Red-S staining and Oil Red-O staining, respectively. (e) SSEA-4^neg^ Saos-2 osteosarcoma cells and SSEA-4^+^ Well5 osteosarcoma cells were exposed to different chemotherapeutic agents for 48 hours, following which apoptosis was measured by flow cytometry (left panel). Data are expressed as means ± SDs (**P* < 0.05, ***P* < 0.01). (f) Viable cell numbers of Well5 and Soas-2 cells after treatment with different concentrations of chemotherapeutic agents for 48 hours. Data are presented as means ± SDs. (g) SSEA-4^+^ cell frequency in primary samples (L1031) or their xenografts was measured by flow cytometric assay before and after one round of chemotherapy.

**
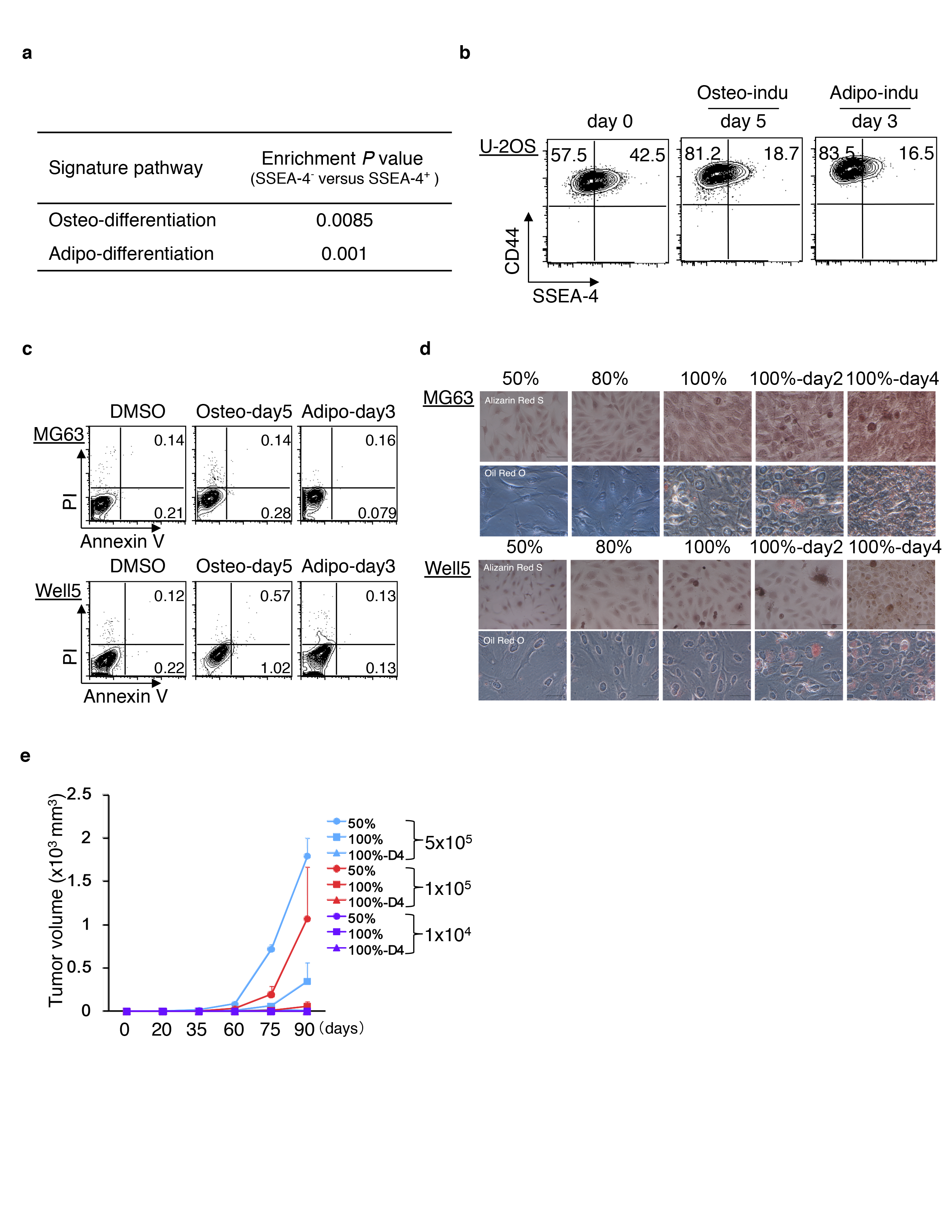
**

**Supplementary Figure 3. SSEA-4^+^ TICs Decrease along with Mesenchymal Differentiation of Osteosarcoma Cells.**

(a) *p* values for the enrichment of signature pathways as indicated, drawn from the analysis of 1491 differentially expressed genes of SSEA-4^-^ cells versus SSEA-4^+^ cells (FDR<0.05). (b) SSEA-4^+^ cell frequency decreased in U-2OS cells undergoing osteogenic or adipogenic differentiation, as measured by flow cytometry. (c) Measurement of apoptosis of MG63 and Well5 cells after osteogenic or adipocytic differentiation induction for 3-5 days. See also Fig. 3d. (d) Extended culture of Well5 and MG63 cells post-confluence induces osteogenic or adipocytic differentiation, as indicated by Alizarin Red and Oil Red-O staining, respectively (scale bar=100μm). See also Fig. 3f. (e) Growth curves of tumorigenic xenografts initiated by the inoculation of Well5 cells collected from different culture conditions as indicated. Data are presented as means ± SDs. See also Fig. 3g.

**
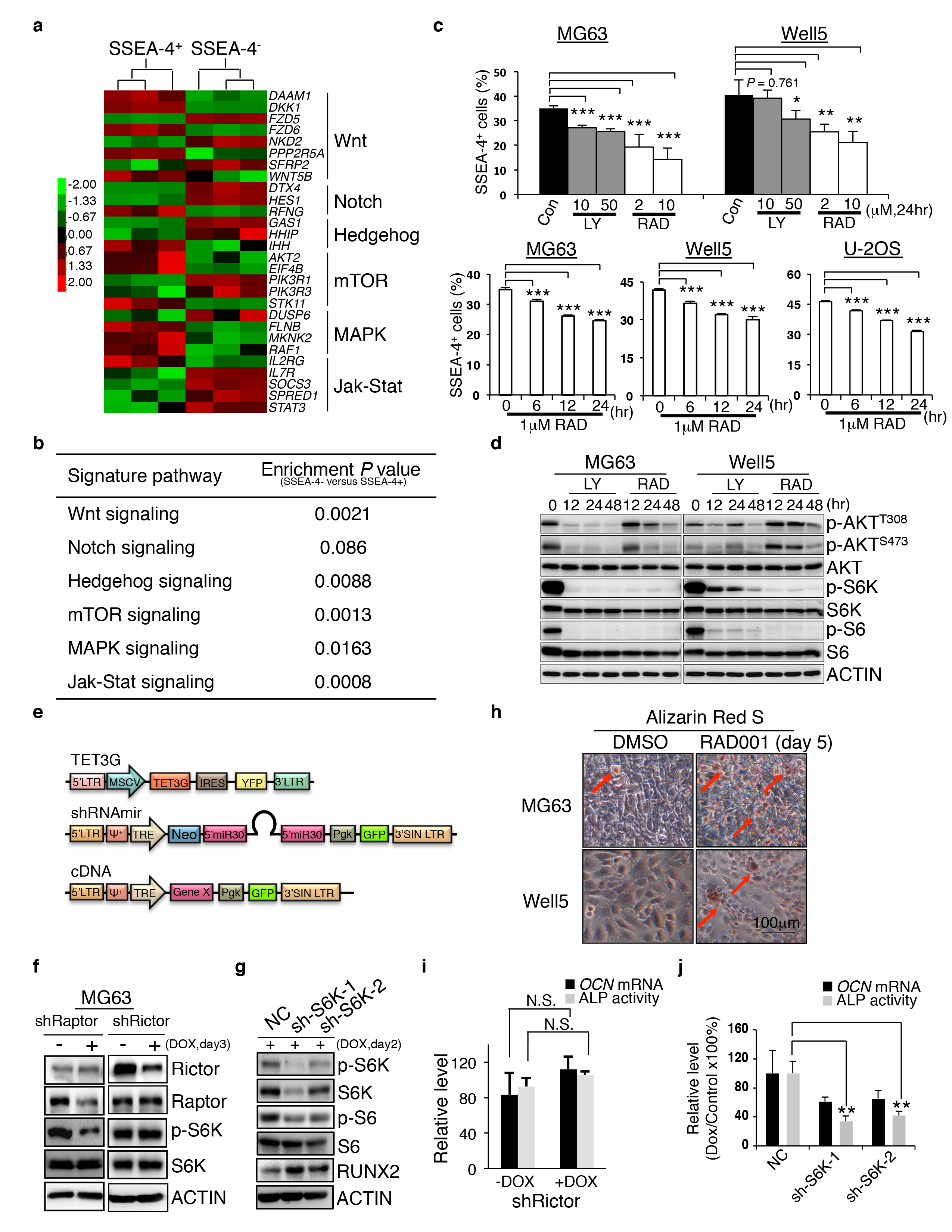
**

**Supplementary Figure 4. Identification of Candidate Pathways that Maintain the SSEA-4^+^ TIC Pool.**

(a) Heatmap shows the expression levels of the signature gene sets that indicate the active status of individual pathways in W0823-derived SSEA-4^+^ and SSEA-4^-^ osteosarcoma cells. (b) The *P* values for the enrichment of signature pathways as indicated, drawn from the analysis of 1491 differentially expressed genes of SSEA-4^-^ cells versus SSEA-4^+^ cells (FDR<0.05). (c) PI3K inhibitor (LY) or mTOR inhibitor (RAD) reduces the frequency of SSEA-4^+^ cells in MG63, Well5 and U-2OS cells, as measured by flow cytometric analysis. Results are expressed as means ± SDs (**P* < 0.05, ***P* < 0.01, ****P* < 0.001). (d) LY and RAD001 inhibit AKT-mTOR activity in osteosarcoma cells, as in (c). (e) Diagrams of the retroviral vectors used for incorporating a Dox-inducible system into osteosarcoma cells. (f-g) Knockdown efficiency of shRaptor, shRictor and shS6K was examined by Western blotting. (h) MG63 and Well5 cells were treated with DMSO or RAD001 for 5 days, and positive Alizarin-staining foci (indicated by arrows) were observed by microscopy. (i) ALP activity and *OCN* mRNA levels were measured in MG63 cells with or without Rictor knockdown. N.S., not significantly different. (j) ALP activity and *OCN* mRNA levels were measured in MG63 cells with or without S6K knockdown (***P* < 0.01, ****P* < 0.001).

**
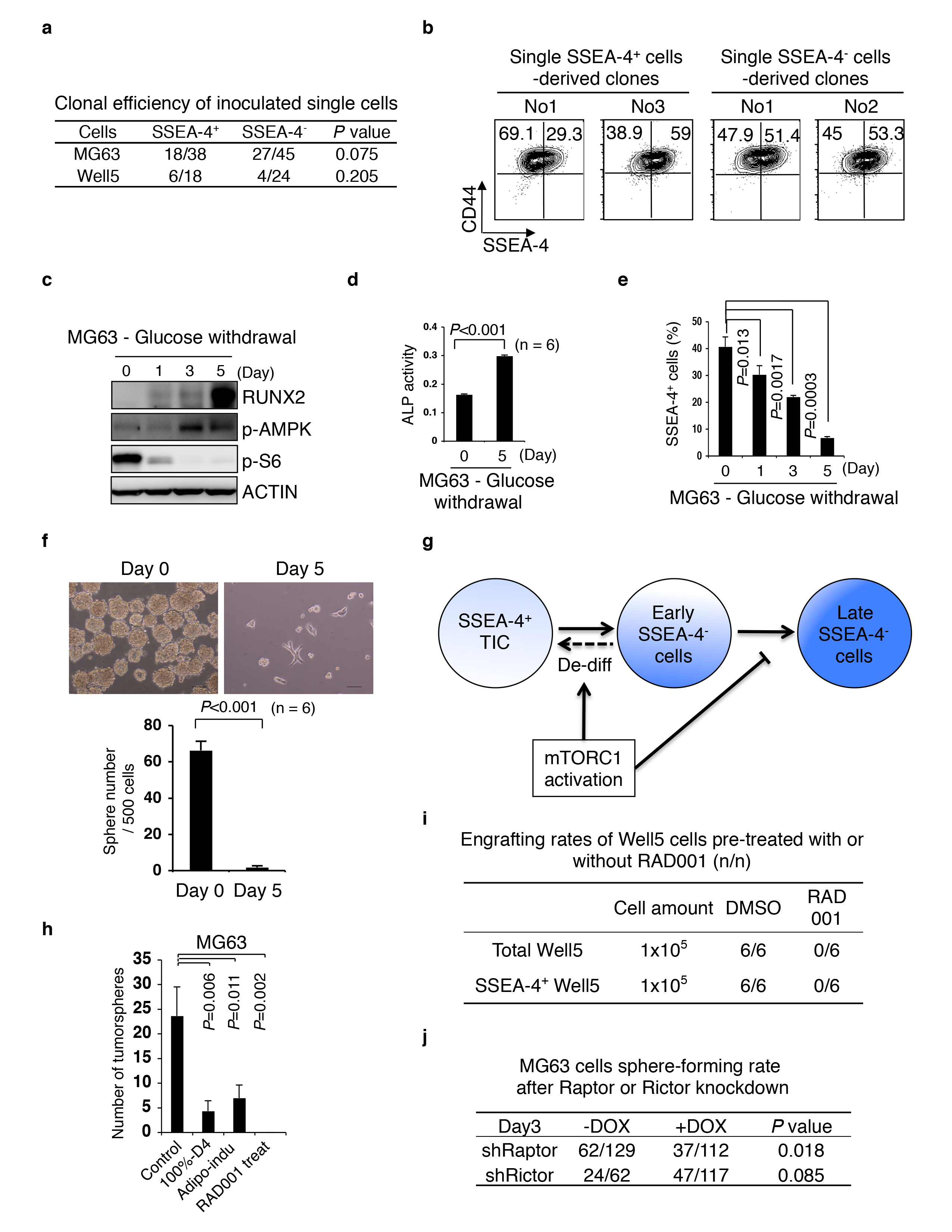
**

**Supplementary Figure 5. mTOR Activity Regulates the Dedifferentiation versus Differentiation Status of SSEA-4^-^ Osteosarcoma Cells.**

(a) The clonal efficiency of single SSEA-4^+^, SSEA-4^-^ MG63 and Well5 cells after inoculation into 96-well plates supplemented with fresh medium. (b) Flow cytometric analysis of colonies of cells from (a). (c-f) Glucose withdrawal from the culture medium activated AMPK and inhibited mTOR activity (c), raised ALP level (d), reduced SSEA-4^+^ cell frequency (e), and diminished tumorsphere-forming capacity (f) of *in vitro*-cultured MG63 cells in a time-dependent manner. (g) The differentiation hierarchy of SSEA-4^+^ TICs and progeny is modulated by mTORC1. (h) The tumorsphere-formation potential of MG63 cells after exposure to different treatments as indicated. Results are expressed as means ± SDs. (i) Xenografting rates of total or SSEA-4^+^ Well5 cells pretreated with PBS or RAD001 (*P* = 0.002 for DMSO versus RAD). (j) The effect of Dox-induced Raptor or Rictor knockdown on the tumor-sphere formation potential of *in vitro* cultured MG63 cells.

**
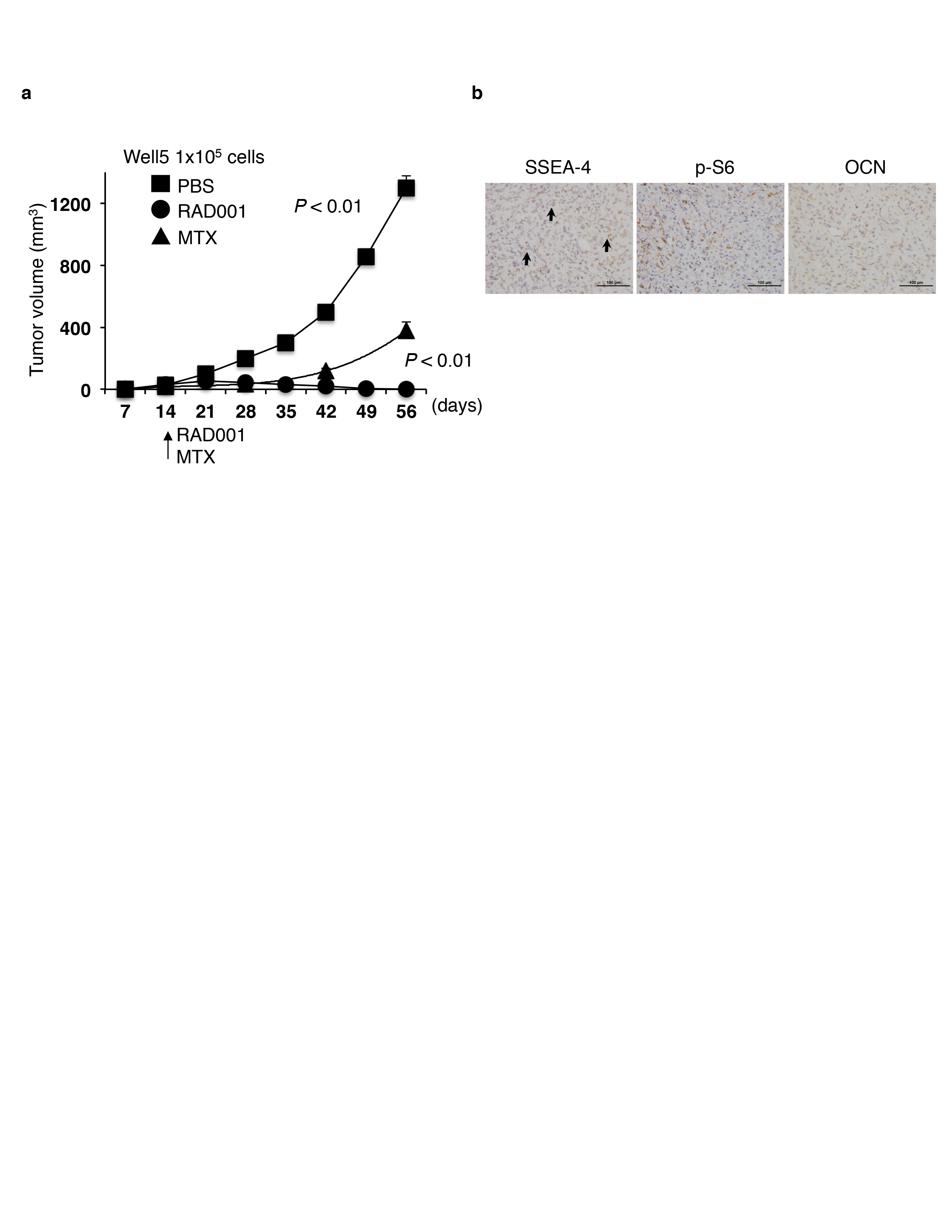
**

**Supplementary Figure 6. Reduction in mTOR Activity Decreases the Chemo-resistance of SSEA-4^+^ TICs by Promoting Osteogenic Maturation.**

(a) Well5 cells were inoculated into NOD/SCID mice which were then treated with PBS, RAD001 or 50 mg/kg methotrexate (MTX) for the indicated times. The growth curves were monitored. (b) Immunohistochemical staining of SSEA-4, S6K and OCN in chemotherapy-treated xenografts (see also Fig. 6e).

**
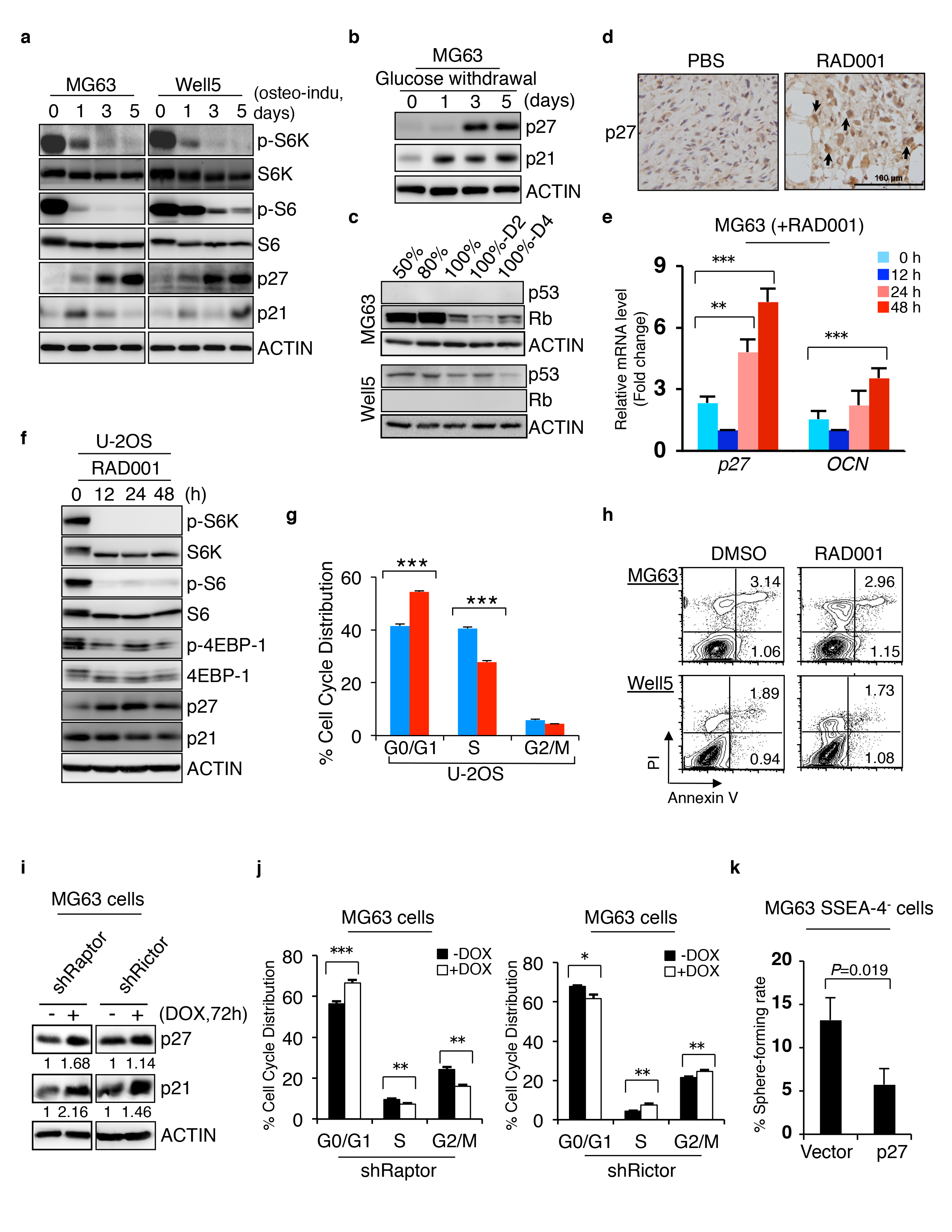
**

**Supplementary Figure 7. mTOR Inactivation Upregulates p27 to Induce Cell-cycle Exit and Terminal Osteogenic Differentiation in Osteosarcoma Cells.**

(a-b) Western blot assays for p21 and p27 levels in MG63 and Well5 cells that were undergoing osteogenic differentiation by a specific inducer (a) or glucose withdrawal (b). (c) Western blot assays of the p53 and Rb levels along with cell confluence-induced mesenchymal differentiation of osteosarcoma cells. (d) Immunohistochemical staining of p27 in xenografts recovered from the PBS group or RAD001 group as in (Fig. 6g). Scale bars represent 100 μm. (e) RAD001 upregulates *p27* and *OCN* mRNA levels in MG63 cells, as assayed by real-time RT-PCR. Data presented are means ± SDs (**P* < 0.05, ***P* < 0.01, ****P* < 0.001). (f) Western blot assay for mTORC1 signaling pathway in U-2OS cells that were treated with 10μM RAD001 for various lengths of time. (g) Cell-cycle status of RAD001-treated U-2OS cells. Data presented are means ± SDs (****P* < 0.001). (h) Apoptotic analyses of MG63 and Well5 cells exposed to DMSO or 10 μM RAD001 for 5 days. (i-j) Western blot assay for the p27 and p21 levels (i) and cell-cycle analysis (j) of MG63 cells with or without Dox-induced Raptor or Rictor knockdown. (k) p27 overexpression further decreases the tumorsphere-forming potential of SSEA-4^-^ MG63 cells.

**
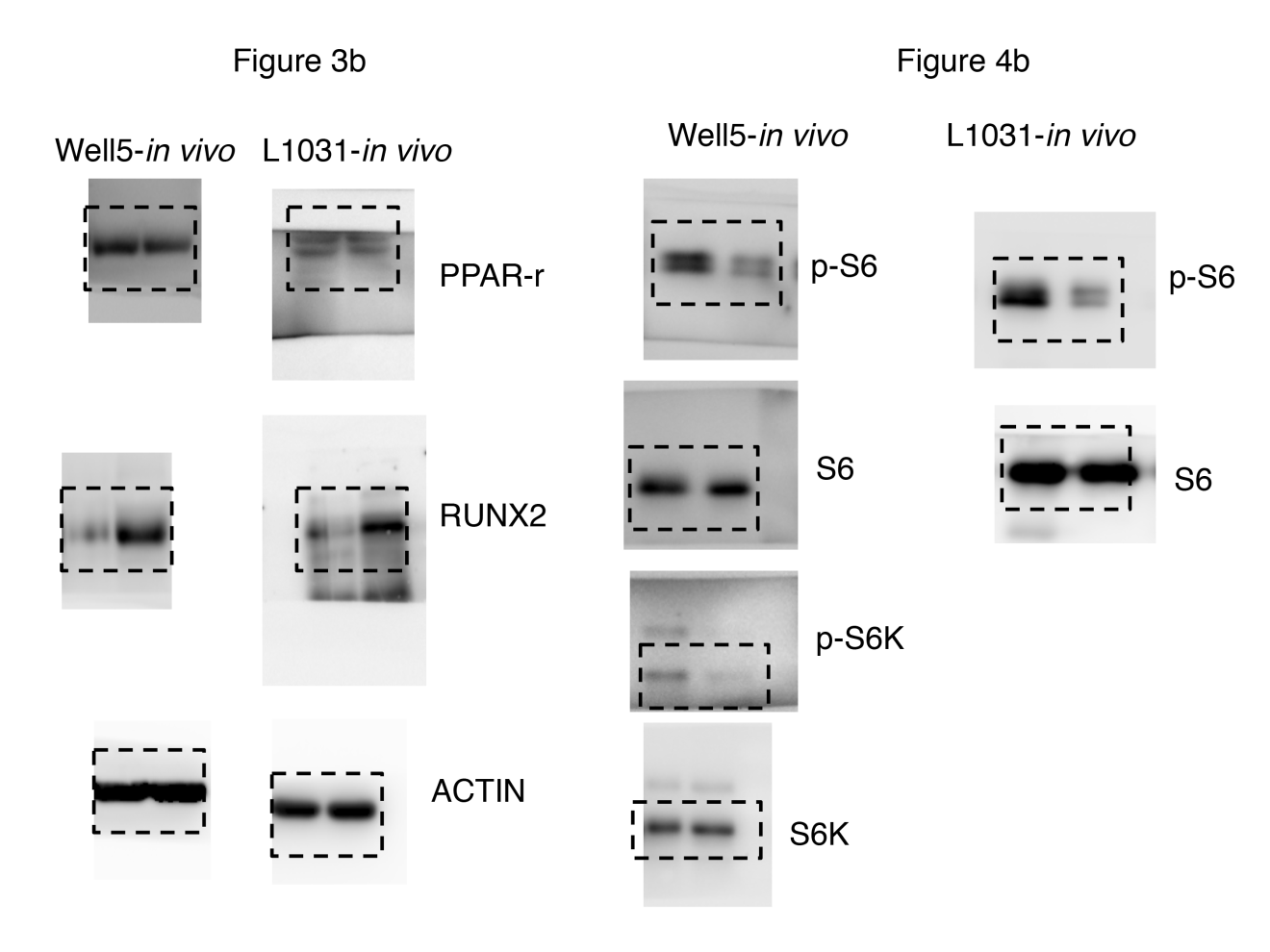

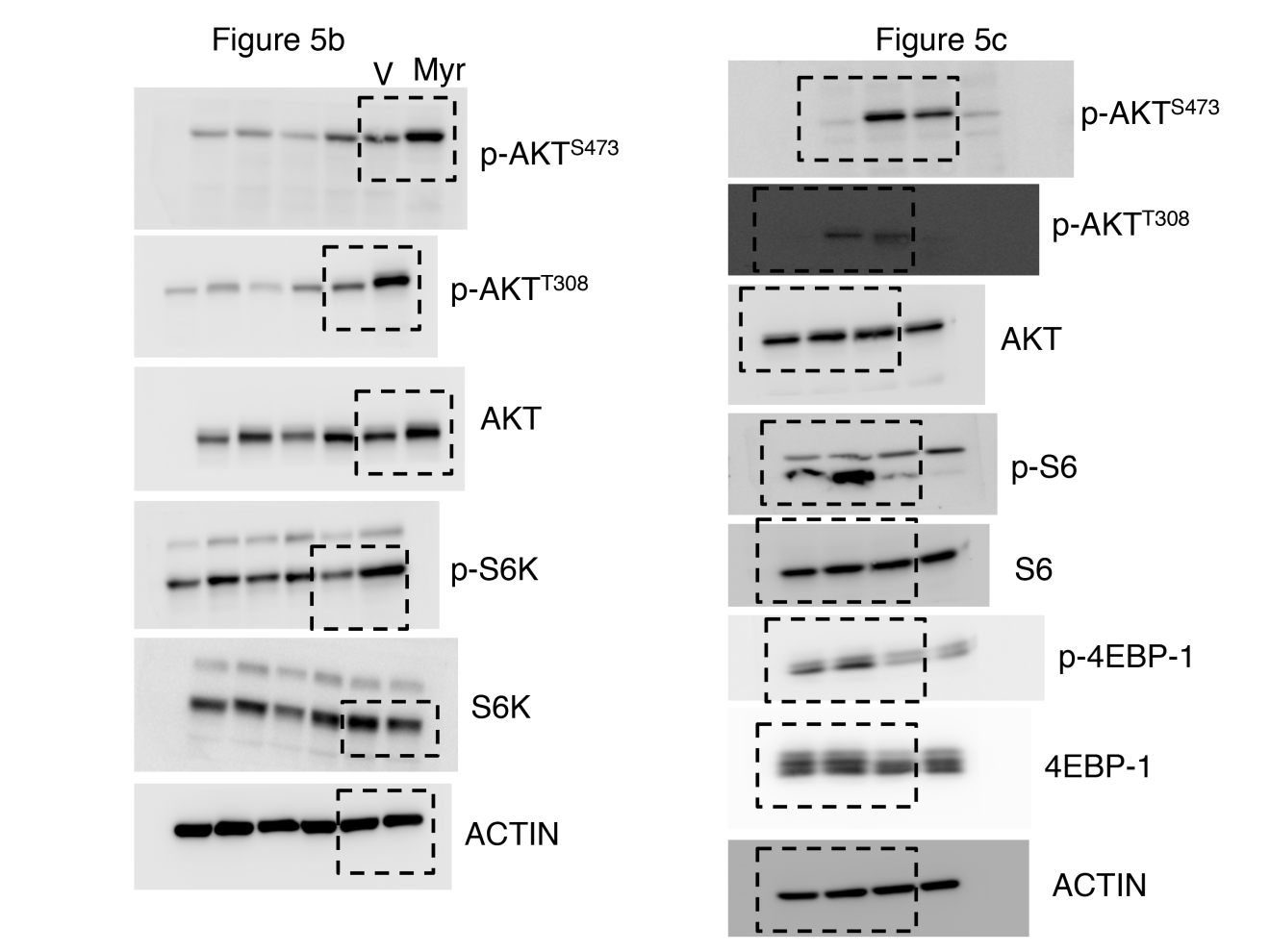

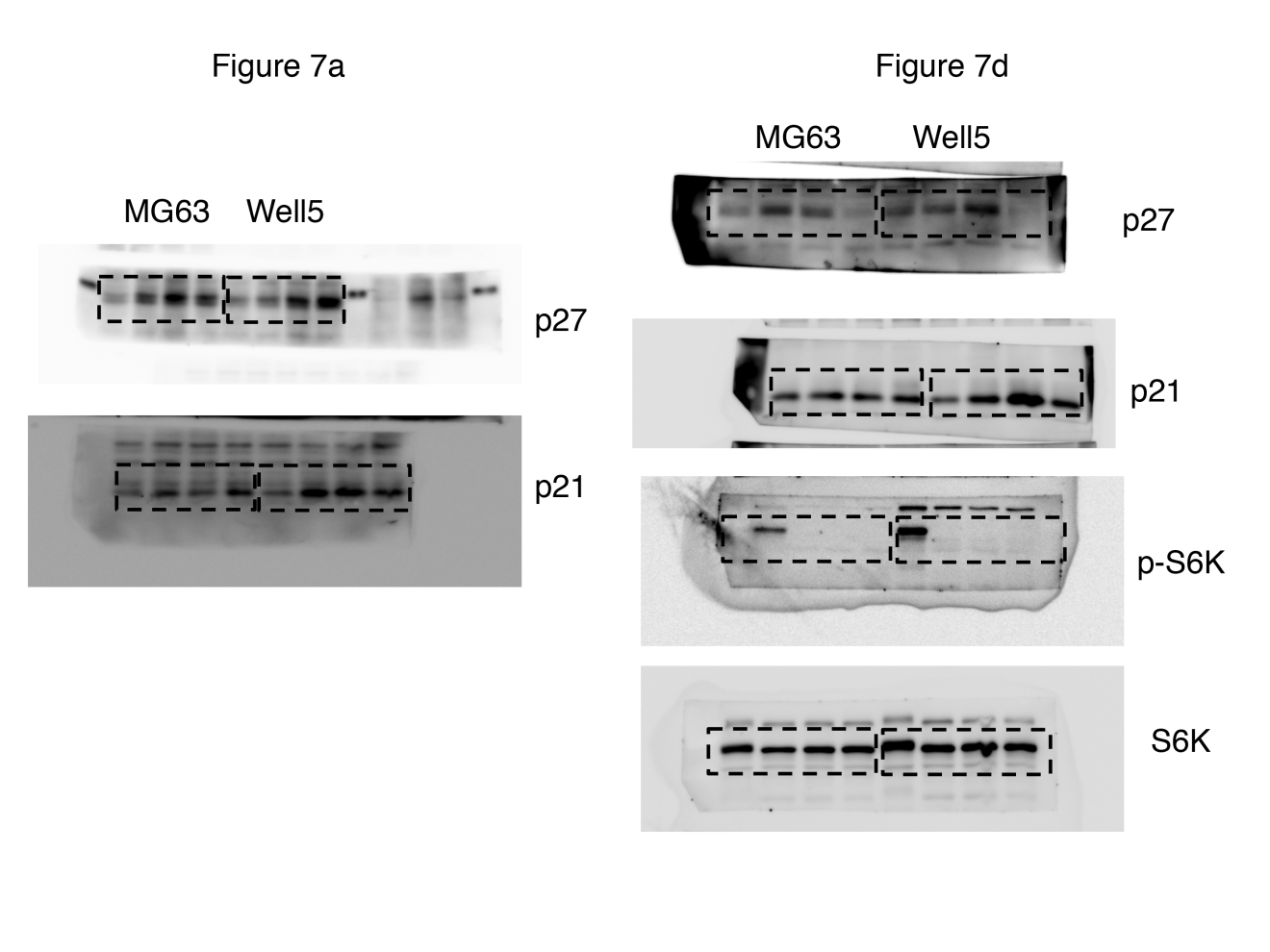
**

**Supplementary Figure 8. Full-length Blots for Figures 3b, 4b, 5b, 5c, 7a and 7d.**

**Supplementary Experimental Procedures**

**Animals**

NOD/SCID mice were purchased from Shanghai SLAC Laboratory Animal Co., Ltd., and were fed and maintained in accordance with the institutional guidelines for animals of Shanghai Jiao Tong University School of Medicine. The numbers of NOD/SCID mice used are indicated in the figures or the corresponding figure legends. All animal experiments were carried out in accordance with guidelines approved and provided by the Laboratory Animal Resource Center of Shanghai Jiao Tong University School of Medicine.

**Culturing of Human Osteosarcoma Cell Lines and Primary Osteosarcoma Cells**

Human osteosarcoma cell lines MG63, SAOS-2 and U-2OS were cultivated in DMEM (Invitrogen, Grand Island, USA) supplemented with 10% FBS. For establishing *in vitro* cultures of primary osteosarcoma cells, tumor samples were minced and further digested into single cell suspensions by collagenase. Cells were then inoculated into 6-well plate and maintained in DMEM supplemented with 10% FBS. Adherent osteosarcoma cells were split and passaged when cell growth reached 80-90% confluence.

**Antibodies and Reagents**

Phycoerythrin (PE)-conjugated and allophycocyanin (APC)-conjugated anti-human/mouse SSEA-4 monoclonal antibodies (IgG3 isotype), mouse IgG_3_ isotype control antibody, PE-conjugated anti-SSEA-1 and anti-SSEA-3 antibodies were purchased from R&D Systems (Minneapolis, USA). All other flow cytometric antibodies were purchased from BD PharMingen (New Jersey, USA), eBiosciences (San Diego, USA), Santa Cruz Biotechnology (Santa Cruz, USA), and Miltenyi Biotec (Leiden, the Netherlands). The majority of immunoblotting antibodies were purchased from Cell Signaling Company (Danvers, USA) and Santa Cruz Biotechnology. LY294002 and SU5402 were purchased from Calbiochem (San Diego, USA). RAD001 was a gift from Novartis (Basel, Switzerland).

**Western Blotting, Immunofluorescent Microscopic Inspection and FISH**

Cells were washed twice with ice-cold PBS and lysed in whole-cell lysis buffer containing 10% SDS. Lysates were resolved by SDS-PAGE and were subsequently transferred to PVDF membranes (GE Healthcare, Buckinghamshire, UK). Membranes were sequentially probed with primary and secondary antibodies. The signals were produced using Immobilon Western kit (Millipore, Billerica, USA), and were visualized using a chemiluminescence detection system (LAS-4000, FUJIFILM). Immunofluorescent staining was performed on either cytospins of single cell suspensions or adherent cultures of human osteosarcoma cells. The probe sets for fluorescence in situ hybridization (FISH) assay of *p53* and *Rb* were purchased from Abbott laboratories (Illinois, USA) and GPmedical (Beijing, China). All probes were labeled and all FISH data were analyzed according to the manufacturers’ protocols.

**RAD001 Treatment**

One-50×10^5^ SSEA-4^+^ cells from different resources were inoculated into 6 to 8 week-old female NOD/SCID recipients. The oral administration of PBS or 5 mg/kg RAD001 began 2 days or 14 days after inoculation. NOD/SCID recipients were treated every other day and tumor volumes were measured weekly.

**Tumorsphere-forming Assay**

Single osteosarcoma cells were inoculated into 96-well plates and cultured in serum-free DMEM-F12 (1:1) medium (Invitrogen) supplemented with 20 ng/ml FGF (R&D Systems), 20 ng/ml EGF (R&D Systems), B27 (1:50) (Invitrogen), N2 (1:100) (R&D Systems), and 10 ng/ml LIF (Millipore) for 3 to 5 weeks.

**Vectors, cDNAs, shRNAs and siRNAs**

YFP^+^ MG63 or Well5 cells constitutively expressing TET3G trans-activator were infected with pTRE3G-GFP, pTRE3G-Myr-AKT-GFP or pTRE3G-p27-GFP lentiviral vectors and sorted by FACS for YFP^+^GFP^+^ cells that responded to Dox. Osteosarcoma cell lines that expressed Dox-inducible Raptor-, Rictor-, S6K-, p27-shRNAs, or control sequences were generated in a similar manner. siRNA oligonucleotides against p27 were transfected using Lipofectamine 2000. Luciferase-targeted oligonucleotides were used as controls.

**Induction of Osteogenic or Adipogenic Differentiation of Osteosarcoma Cells**

Induction of differentiation of osteosarcoma cells was performed following the protocols provided by the vendor (Millipore, Temecula, USA).

**ALP Assay**

ALP was measured using *1-Step*^TM^ PNPP kit purchased from Thermo Scientific (Rockford, IL, USA).
